# Altered Expression of Matrix Metalloproteinases and Their Endogenous Inhibitors in a Human Isogenic Stem Cell Model of Huntington's Disease

**DOI:** 10.3389/fnins.2017.00736

**Published:** 2018-02-05

**Authors:** Swati Naphade, Alexander Embusch, Kuruwitage Lakshika Madushani, Karen L. Ring, Lisa M. Ellerby

**Affiliations:** ^1^The Buck Institute for Research on Aging, Novato, CA, United States; ^2^California Institute of Regenerative Medicine, San Francisco, CA, United States

**Keywords:** Huntington's disease, neural stem cells, matrix metalloproteinases, tissue inhibitors of metalloproteinases, transforming growth factor-β

## Abstract

Huntington's disease (HD) is an autosomal dominant neurodegenerative disorder characterized by a progressive movement disorder, psychiatric symptoms, and cognitive impairments. HD is caused by a CAG repeat expansion encoding a stretch of polyglutamine residues in the N-terminus of mutant huntingtin (mHTT) protein. Proteolytic processing of mHTT yields toxic fragments, which cause neurotoxicity and massive neuronal cell death predominantly in the striatum and cortex. Inhibition of mHTT cleavage reduces neuronal toxicity suggesting mHTT proteolysis contributes to HD pathogenesis. A previously conducted unbiased siRNA screen in our lab for known human proteases identified matrix metalloproteinases (MMPs) as modifiers of mHTT proteolysis and toxicity. To further study MMP activation in HD, isogenic HD, and control corrected (C116) neural stem cells (NSCs) prepared from HD patient-derived induced pluripotent stem cells were used to examine the role of MMPs and their endogenous inhibitors in this highly relevant model system. We found altered expression of MMP-2 and MMP-9 (gelatinases), MMP-3/10, and MMP-14, activity in HD-NSCs when compared to control C116-NSCs. Dysregulation in MMP activity was accompanied with concomitant changes in levels of endogenous inhibitors of MMPs, called tissue inhibitors of matrix metalloproteinases (TIMPs). Specifically, we observed decreased levels of TIMP-1 and TIMP-2 in HD-NSCs, suggesting part of the altered expression and activity of MMPs is due to lower abundance of these endogenous inhibitors. Immunofluorescence analysis revealed increased MMP/TIMP localization in the nucleus or aggregates of HD-NSCs, suggesting potential interaction with mHTT. TIMP-1 was found to associate with mHTT aggregates in discrete punctate structures in HD-NSCs. These events collectively contribute to increased neurotoxicity in HD. Previous characterization of these NSCs revealed transforming growth factor beta (TGF-β) pathway as the top dysregulated pathway in HD. TGF-β was significantly upregulated in HD-NSCs and addition of TGF-β to HD-NSCs was found to be neuroprotective. To determine if TGF-β regulated MMP and TIMP activity, C116- and HD-NSCs were exogenously treated with recombinant TGF-β. TIMP-1 levels were found to be elevated in response to TGF-β treatment, representing a potential mechanism through which elevated TGF-β levels confer neuroprotection in HD. Studying the mechanism of action of MMPs and TIMPs, and their interactions with mHTT in human isogenic patient-derived NSCs elucidates new mechanisms of HD neurotoxicity and will likely provide novel therapeutics for treatment of HD.

## Introduction

Primarily characterized as a movement disorder, Huntington's disease (HD) is an autosomal-dominant neurodegenerative disorder with cognitive decline, chorea, and emotional disturbances as the disease progresses. Marked striatal atrophy accompanied with selective degeneration of medium spiny neurons represent classical pathological hallmarks of HD. HD is caused by an expanded CAG triplet repeat in the huntingtin (HTT) gene that encodes a stretch of polyglutamine residues which renders the mutant huntingtin (mHTT) protein prone to aggregate formation. mHTT is susceptible to proteolysis at the N-terminus with the resulting short N-terminal fragments contributing to cellular toxicity by inducing apoptotic cell death. Truncated N-terminal mHTT fragments have been reported in human HD postmortem tissue and mouse models of HD (Mende-Mueller et al., 2001; Wellington et al., 2002; Wang et al., 2008). Furthermore, inhibition of mHTT cleavage reduced toxicity both *in vitro* and *vivo* (Wellington et al., 2000; Gafni et al., 2004; Graham et al., 2006), indicating an important role for mHTT proteolysis in HD pathogenesis. In order to identify critical proteases that directly cleave mHTT, an unbiased western blot-based siRNA screen for 514 known human proteases was conducted (Miller et al., 2010). This screen confirmed 11 proteases that, when silenced, reduced toxic N-terminal HTT fragment formation. Interestingly, three of these eleven modifiers of HTT proteolysis and toxicity belonged to the matrix metalloproteinase (MMP) family (MMP-10, -14, and -23B).

MMPs are Ca^2+^ dependent, zinc-containing proteolytic enzymes. At least 25 members of the MMP family have been identified in humans so far, and they exhibit different substrate specificity and domain organizations categorized into collagenases, stromelysins, gelatinases, membrane-type MMPs (MT-MMPs), matrilysins, and other MMPs (Nagase et al., 2006; Table 1). MMPs are mostly secreted into the extracellular space, except for MT-MMPs that are transmembrane proteases. MMPs are first produced as inactive zymogens and are activated by other proteases (or MMPS) or free radicals (Ra and Parks, 2007). They occupy central roles in several normal physiological processes, including, stem cell differentiation, proliferation, migration, wound repair, angiogenesis, and apoptosis (Malemud, 2006). Although altered MMP expression has been observed in several neurodegenerative diseases (Brkic et al., 2015), including Alzheimer's disease (AD) (Lorenzl et al., 2003b; Lim et al., 2011), Parkinson's disease (PD) (Lorenzl et al., 2002), and amyotrophic lateral sclerosis (ALS) (Lim et al., 1996; He et al., 2013), the exact contribution of MMPs to the pathogenesis of diseases remains unclear. MMP activity is tightly regulated *in vivo* by endogenous inhibitors such as tissue inhibitors of metalloproteinases (TIMPs) (Brew and Nagase, 2010). The mammalian TIMP family presently consists of four members (TIMP-1 to -4). TIMPs inhibit active forms of MMP by binding to the Zn^2+^ cation in the MMP catalytic domain. Studies indicate that TIMPs also serve MMP-independent functions that help modulate cell proliferation, apoptosis, and synaptic plasticity (Brew and Nagase, 2010).

**Table 1:**
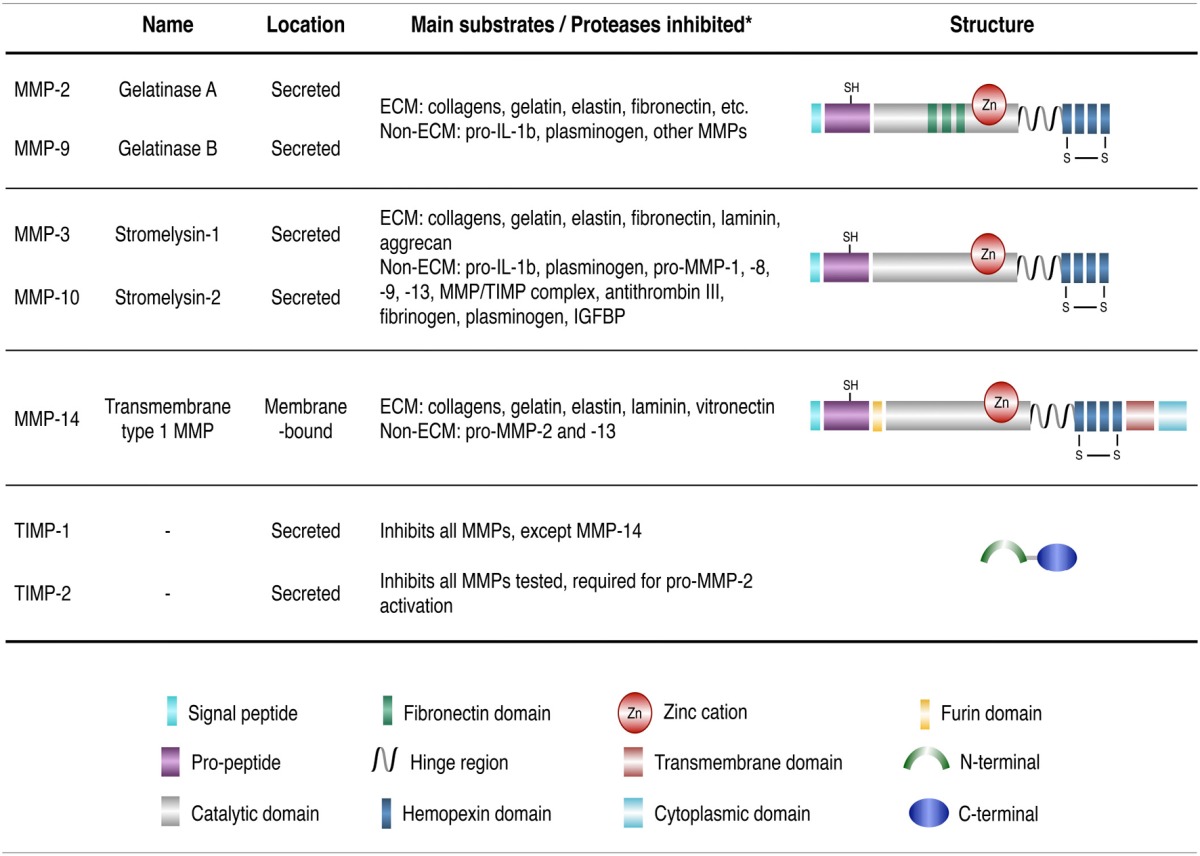
Classification of MMPs and TIMPs.

ECM, extracellular matrix. ^*^References: 1. Crocker et al. (2004). 2. Kapoor et al. (2016).

Previous studies from our lab demonstrated an upregulation of MMP-10 and MMP-14 in Hdh^111Q/111Q^ mouse striatal cells compared to the Hdh^7Q/7Q^ cells (Miller et al., 2010). Additionally, MMP-10 was shown to directly cleave HTT into toxic N-terminal fragments. siRNA mediated knockdown of MMP-10 and MMP-14, or overexpression of TIMPs, in Hdh^111Q/111Q^ blocked caspase-3/7 activity, a direct measure of cellular apoptosis. Furthermore, treatment with NNGH, a broad spectrum MMP inhibitor, resulted in a dose-dependent inhibition of caspase-3/7 activity. These data unequivocally show that MMP inhibition ameliorates mHTT-induced striatal toxicity. Elevated MMP activity was also observed in the striatum of older R6/2 and YAC128 HD mouse models, suggesting that MMPs exhibit specific spatial and temporal patterns of expression. Finally, reducing MMP activity significantly improved motor performance in the HD Drosophila model. These studies underscore the need for further investigation into the role of MMPs and TIMPs in HD in order to design effective therapies.

To this end, we characterized MMP and TIMP expression in an induced pluripotent stem cell (iPSC)-derived neural stem cell (NSC) model developed previously in our lab (An et al., 2012). Briefly, patient-derived HD-iPSCs (72Q/19Q) were genetically corrected by replacing the expanded CAG repeat (72Q/19Q) with a normal repeat using targeted homologous recombination. The corrected “C116-iPSCs” (21Q/19Q) retained characteristics of pluripotent stem cells. The merit of this approach is that both the C116- and HD-iPSC lines are isogenic (thus, differences in gene expression can be solely attributed to the length of the CAG repeat) and allow for patient-specific modeling of HD. Previous characterization using differential gene expression and pathway analysis revealed that more than 4,000 genes were differentially expressed in HD-NSCs in contrast to only about 400 genes in HD-iPSCs. These findings indicated that HD-associated cellular and molecular phenotypes were evident only in differentiated HD-NSCs but not in HD-iPSCs (Ring et al., 2015), making NSCs an attractive human HD cellular model.

Our results demonstrate altered MMP expression and cellular localization in HD-NSCs as compared to C116-NSCs. Both TIMP-1 and TIMP-2 were found to be downregulated in HD-NSCs. Previous genomic analysis of HD-and corrected C116-NSCs revealed increased upregulation of TGF-β expression in HD-NSCs. This upregulation was shown to be neuroprotective, thereby reflecting a possible compensatory attempt to re-establish CNS homeostasis in HD (Ring et al., 2015). However, the mechanism remains unknown. In this study, we show that upregulation of TIMP-1 expression in response to TGF-β represents a potential mechanism through which TGF-β confers neuroprotection in HD-NSCs.

## Materials and methods

### RNA-Seq analysis

**Figure 1:**
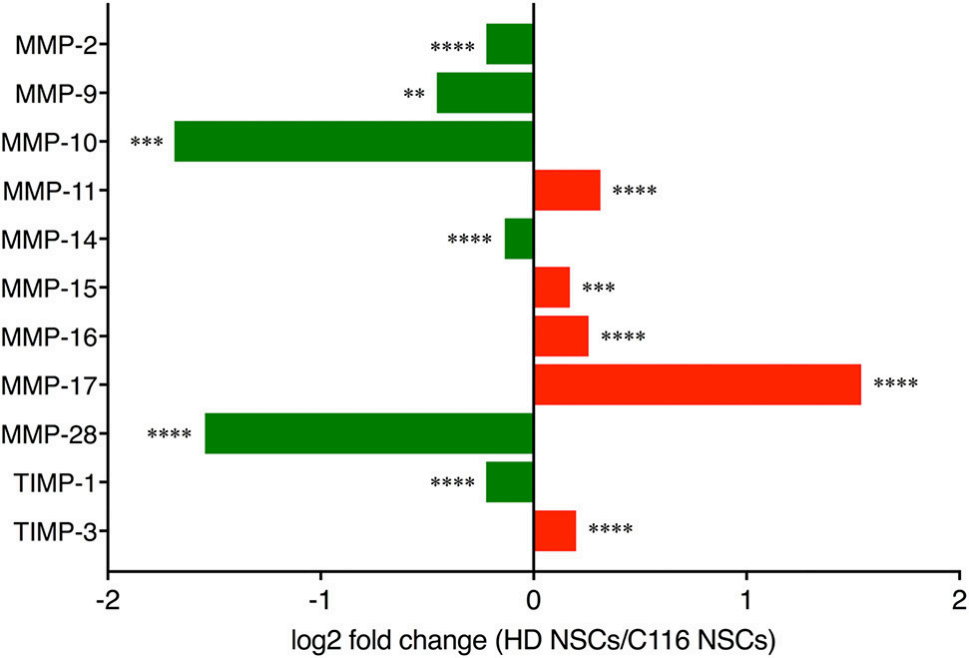
RNA-Seq analysis. RNA-Seq analysis of MMP and TIMP expression in HD- vs. corrected C116-NSCs via IPA analysis reveals a dysregulated MMP/TIMP axis in HD-NSCs (^**^p < 0.01; ^***^ *p* < 0.001; ^****^ *p* < 0.0001). Data mined from Ring et al. (2015).

RNA-Seq analysis was performed as described previously and Figure 1 was generated using this data set (Ring et al., 2015). Briefly, total RNA was purified from iPSCs and NSCs using the RNeasy Mini kit (Qiagen). A Qiacube instrument was used to extract the RNA using the RNase Micro protocol. Subsequent RNA-Seq by Illumina library preparation was performed on an Illumina Hiseq 2000 sequencer. Resulting data was analyzed using enrichment and networking analysis and hierarchical clustering analysis. The accession number for the RNA-Seq data used is GEO: GSE74201.

### Targeted correction of the expanded HTT gene in HD-iPSCs

Patient-derived HD-iPSCs (72Q/19Q) were corrected using targeted homologous recombination, resulting in the reduction of the expanded HTT gene to normal 21 polyglutamine repeats, as described previously (An et al., 2012). Correctly targeted clones (21Q/19Q) were selected using G418 selection and verified using western and Southern blot analysis. The corrected C116-iPSCs comprised of a homogeneous cell population and retained characteristics of pluripotent stem cells (An et al., 2012).

### Cell culture

NSCs were generated from C116- and HD-iPSC lines using STEMdiff Neural Induction Medium (Stem Cell Technologies) (Ring et al., 2015) and characterized (An et al., 2012) as described previously. 6 cm dishes or 6-well plates (Corning, Nunclon Delta Surface) were coated with Matrigel (1:60; BD Corning) for 1 h. NSCs were plated and cultured in Neural Proliferation Medium (NPM) in humidified incubator under 37°C, 5% CO_2_. NPM was prepared using Neurobasal medium supplemented with 1X B-27 supplement (Life Technologies), 2 mM L-Glutamine, 100 U/mL penicillin, 100 μg/mL streptomycin, 10 ng/mL human Leukemia Inhibitory Factor (LIF) (Peprotech, 300-05), and 25 ng/mL human basic Fibroblast Growth Factor (bFGF) (Peprotech, 100-18B).

### TGF-β treatment

C116- and HD-NSCs were treated with recombinant human TGF-β1 (Peprotech, AF-100-21C) reconstituted in NPM at 10 or 20 ng/mL concentration for 1 or 24 h. Untreated NSCs served as controls.

### Western blot analysis

NSCs were harvested in M-PER Mammalian Protein Extraction Reagent (Pierce) with cOmplete Mini EDTA-free protease inhibitor (1 tablet/10 mL) (Roche). Whole-cell lysates from NSCs were sonicated with a 5 second pulse followed by a 5 second rest (X 5 times) at 40% amplitude. Samples were centrifuged at 14,000 rpm at 4°C for 20 min, and supernatant was collected and stored at −20°C. Protein concentrations were estimated using the BCA assay (Pierce). Cell lysates were denatured under reducing conditions by boiling 10–20 μg total protein with 1 μL of 1M DTT and 4X LDS sample buffer (Invitrogen) at 95°C for 10 min. SDS-PAGE was performed using NuPage 4-12% Bis-Tris gels (Invitrogen). Gels were run in 1X MES or 1X MOPS running buffer containing 500 μL of antioxidant (Life technologies), and then transferred to 0.45 μm PVDF membrane using 1X NuPage transfer buffer at 20 V for 14 h. Membranes were blocked with 5% non-fat milk in TBS with 0.1% Tween 20 (TBS-T) for 1 h at room temperature (RT), incubated with primary antibody reconstituted in 5% non-fat milk overnight at 4°C, followed by incubation with secondary antibody for 2 h at RT. Blots were washed in TBS-T for 10 min (3X), and developed using Pierce ECL (Thermo Scientific). β-actin or α-tubulin served as loading controls. Densitometry analysis was performed using ImageQuant TL v2005. Western blot analysis was performed to study MMP/TIMP expression at basal levels in C116- and HD-NSCs, and additionally in presence or absence of exogenously added TGF-β. Biological replicates (BR) were used for each experiment. Primary and secondary antibodies used are listed in Supplementary Table 2.

### Gelatin zymography

C116- and HD-NSCs were harvested in lysis buffer (50 mM Tris-HCL (pH 7.6), 150 mM NaCl, 5 mM CaCl_2_, 0.05% Birj-35, 1% Triton X-100, 0.02% NaN_3_), and sonicated as described above. For each sample, 500 μg of sonicated cell lysate was suspended in 500 μL of lysis buffer and 50 μg of Gelatin Sepharose 4B beads (GE healthcare) and placed on rotator for 1 h at 4°C for MMP binding. Suspensions were centrifuged, supernatant was removed, and affinity-bound MMPs were eluted by resuspending the bead complexes in 50 μL elution buffer (10% DMSO in PBS). 20 μg of elute (with 4X LDS sample buffer) was then loaded on a 8% zymogram gel containing 0.1% gelatin (Resolving gel: 1.5 M Tris-HCl (pH 8.8), 0.4% SDS, ddH_2_O, 40% acrylamide/bis-acrylamide, tetramethylethylenediamine (TEMED), 10% ammonium persulfate (APS), and 0.1% gelatin. Stacking gel: 1.0 M Tris-HCl (pH 6.8), ddH_2_O, 40% acrylamide/bis-acrylamide, TEMED, 10% APS). Zymogram gels were run in 1X Tris-Glycine SDS buffer under non-reducing conditions at 150 V for 1 h. Gels were then washed with renaturation buffer (2.5% Triton X-100 in ddH_2_O) for 40 min at 25°C, briefly rinsed with ddH_2_O, and incubated in 50 mL of incubation buffer (50 mM Tris HCl, 0.15 M NaCl, 10 mM CaCl_2_) for 20-72 h at 37°C. Finally, gels were briefly washed with ddH_2_O, stained with 0.05% Coomassie Brilliant Blue solution for 1 h, and de-stained for 1 h at RT. Gels were scanned using an Epson Scanner.

For conditioned media, fresh medium was added to near-confluent cultures and the media was harvested after 3 days of incubation at 37°C and 5% CO_2_. Five hundred microliters of conditioned media was loaded onto Amicon Ultra-0.5 Centrifugal Filter Devices (EMD Millipore, UFC501008) with a molecular weight cut-off of 10 kD. Conditioned media was concentrated to 20 μL by centrifuging the devices at 14,000 × g for 30 min. Concentrated media was recovered by turning the devices upside down in clean tubes and centrifuging the tubes at 1,000 × g for 2 min. 20 μg of elute (with 4X LDS sample buffer) was then loaded on a 8% zymogram gel containing 0.1% gelatin, and gelatin zymography was performed as described above.

### Immunocytochemistry and fluorescence microscopy

C116- and HD-NSCs were plated in Matrigel-coated 8-well glass chamber slides (BD Falcon) (80,000 cells per well). Cells were fixed in 4% paraformaldehyde for 15 min at RT and washed three times with PBS. Cells were permeabilized and blocked with 0.1% Triton X-100, 10% donkey serum in PBS for 30 min at RT. The cells were washed once with PBS and incubated with primary antibody (suspended in 1% BSA in PBS) for 24 h at 4°C in a humid chamber. Slides were then washed with PBS (10 min, 3X), and incubated with secondary antibody (suspended in 1% BSA in PBS) at RT for 90 min in the dark. Finally, slides were washed with PBS (5 min, 3X), and coverslips were mounted with ProLong Gold with DAPI antifade reagent (Life Technologies). Slides were cured 24 h in the dark at RT. Imaging was performed on Nikon Eclipse Ti-U microscope using the Plan Apo λ 20X/0.75 objective. Z-stacks for Supplementary Figure 2 were acquired on the Zeiss LSM 780 confocal microscope using the 63X Zeiss plan-apochromat oil, 1.4 NA, DIC objective. Primary and secondary antibodies used are listed in Supplementary Table 2.

### RT-PCR analysis

Total RNA was isolated from NSCs using ISOLATE II RNA Mini Kit (Bioline). cDNA was prepared from 1 μg of RNA in a total reaction volume of 20 μl using the SensiFAST cDNA synthesis kit (Bioline). RT-PCR reactions were setup in a 384-well format using 2X SensiFAST Probe No-ROX kit (Bioline) and 1 μl cDNA per reaction in a total volume of 10 μl. RT-PCR was performed on the Roche LightCycler 480 instrument. For quantification, the threshold cycle, Ct, of each amplification was determined by using the second derivative maximum method. The 2^−ΔΔCt^ method was used to determine the relative expression levels of each gene normalized against the house-keeping gene β-Actin. Primer-probes used are listed in Supplementary Table 3.

### Statistical analysis

Student's paired *t*-test and ANOVA with Tukey's multiple comparison test was used to study differences in MMP/TIMP expression in C116- and HD-NSCs in presence or absence of recombinant TGF-β. All statistical analysis and graph plotting was performed using PRISM 7 by GraphPad Software (La Jolla, CA, USA). *p* < 0.05 was considered as statistically significant.

## Results

RNA-Seq analysis revealed altered expression of MMPs and TIMPs in HD-NSCs compared to C116-NSCs (Figure 1) indicating a dysregulation of the MMP/TIMP axis in HD-NSCs.

### MMP expression is modulated in HD

#### MMP-3/10 and MMP-14 levels are altered in HD

**Figure 2:**
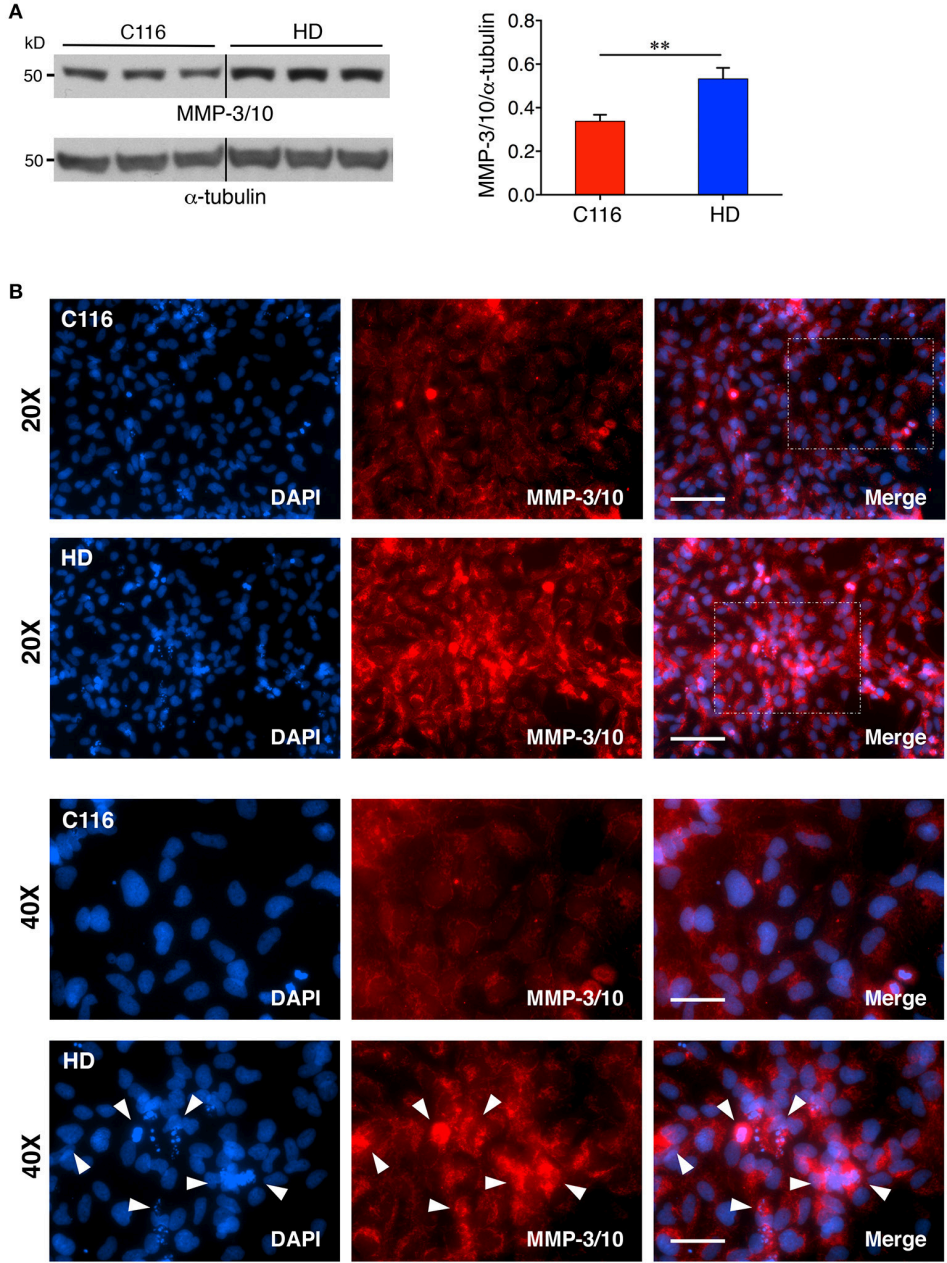
MMP-3/10 expression is elevated in HD-NSCs. **(A)** Western blot analysis of MMP-3/10 reveals a robust increase in expression in HD-NSCs. MMP-3/10 immunoreactivity was quantified by densitometry and normalized for α-tubulin immunoreactivity (*n* = 3 BR, ^**^p < 0.01; *t*-test). Error bars represent *SD*. **(B)** Immunofluorescence analysis confirms increased expression of MMP-3/10 in HD-NSCs, and also shows elevated nuclear presence of MMP-3/10 in HD-NSCs (40X magnified insets) as compared to C116-NSCs. Additionally, increased MMP-3/10 immunoreactivity is observed in apoptotic cells with degenerating nuclei in HD-NSCs (40X magnified inset, white arrowheads). Scale Bars: 20X = 100 μm, 40X = 50 μm.

**Figure 3:**
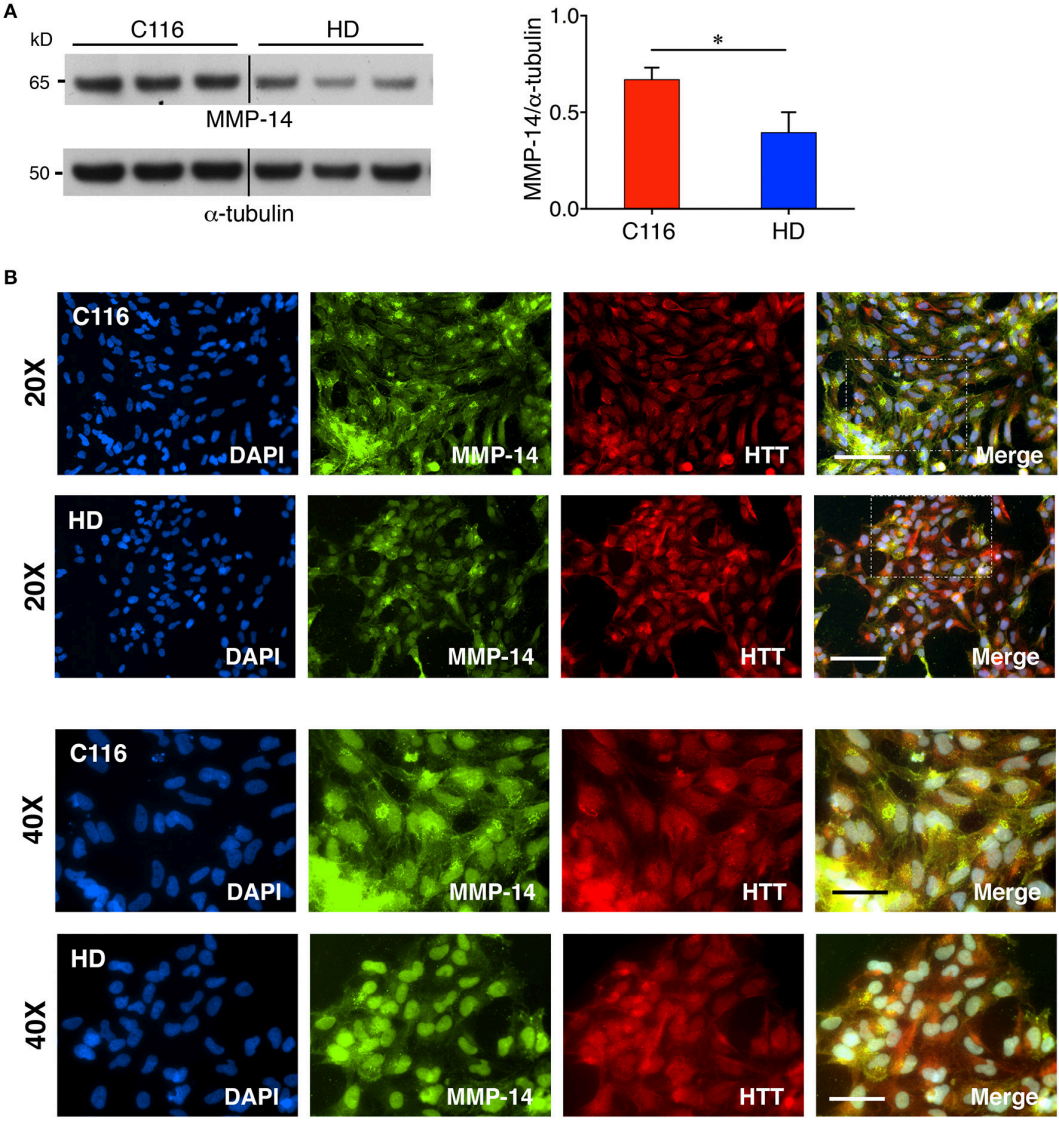
Decreased MMP-14 expression in HD-NSCs. **(A)** Western blot analysis of MMP-14 shows a significantly decreased expression of MMP-14 in HD-NSCs. MMP-14 immunoreactivity was quantified by densitometry and normalized for α-tubulin immunoreactivity (*n* = 3 BR, ^*^p < 0.05; *t*-test). Error bars represent *SD*. **(B)** Immunofluorescence analysis of MMP-14 not only confirms decreased expression in HD vs. C116-NSCs, but also reveals predominantly nuclear expression of MMP-14 in HD-NSCs which possibly associates with nuclear mHTT expression (40X magnified insets). Scale Bars: 20X = 100 μm, 40X = 50 μm.

Our previous studies showed elevated levels of MMP-14 and proteolytically processed active MMP-10 form in mouse striatal Hdh^111Q/111Q^ cells compared to Hdh^7Q/7Q^ cells (Miller et al., 2010). Additionally, MMP-10 was reported to directly cleave HTT. Another recent study reported a direct correlation between increasing MMP-3 levels in cerebrospinal fluid (CSF) and worsening of disease in HD patients (Connolly et al., 2016). To determine if MMP levels are also dysregulated in our human iPSC-derived NSC model, control C116- and HD-NSC lysates were subjected to western blot analysis. MMP-3 and MMP-10 are both stromelysins and share the highest sequence homology (86%) among all MMPs (Bertini et al., 2004), potentially serving redundant biochemical functions. Consistent with our previous findings, MMP-3/10 levels were found to be elevated in HD-NSCs compared to C116-NSCs [Figure 2A (1.6-fold increase), Supplementary Figure 1A (3.6-fold increase), Supplementary Figure 1B (1.4-fold increase)]. Immunofluorescence analysis not only confirmed these findings but also revealed altered localization of MMP-3/10 between the two genotypes (Figure 2B). While MMP-3/10 is predominantly expressed in the cytoplasm in C116-NSCs, robust nuclear expression is also observed in HD-NSCs, which appears to be associated with apoptotic cells (degenerating nuclei) as well (white arrowheads in Figure 2B). Interestingly, MMP-14 levels were decreased in HD-NSCs compared to C116-NSCs (Figure 3). Despite the reduced overall levels detected by western blot analysis (Figure 3A, 1.7-fold decrease) and RT-PCR analysis (Supplementary Figure 1C, 15.4-fold decrease), MMP-14 expression was found to be strongly nuclear in HD-NSCs where it possibly associates with nuclear HTT immunoreactivity (Figure 3B).

#### MMP-2 and MMP-9 (gelatinases) expression in HD

**Figure 4:**
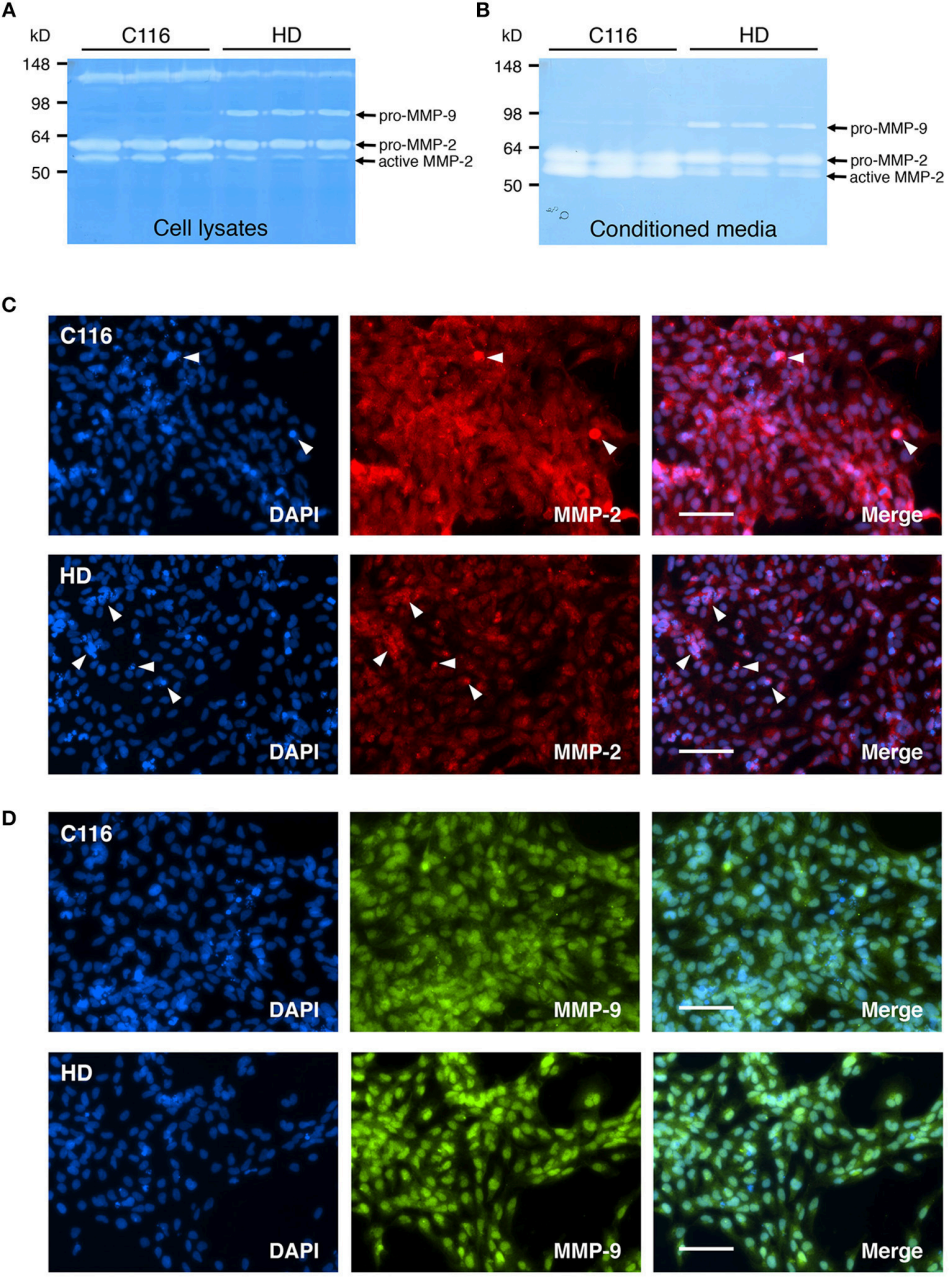
Expression of MMP-2 and MMP-9 (Gelatinases) is altered in HD-NSCs. Gelatin zymogram of C116- and HD-NSC lysates **(A)** shows increased activity of pro-MMP-9 in HD-NSCs, which is also extracellularly secreted into the conditioned media **(B)**. In contrast, decreased production of active-MMP-2 is observed in samples prepared from HD-NSCs when compared to C116-NSCs. Immunofluorescence analysis using anti-MMP-2 **(C)** and anti-MMP-9 **(D)** antibodies confirms these findings and reveals strictly nuclear localization of both MMP-2 and -9 in HD-NSCs. Strongly increased MMP-2 expression was also found in association with apoptotic cells (white arrowheads in **C**). Scale Bars: 100 μm.

Altered levels of MMP-2 (Gelatinase A) and MMP-9 (Gelatinase B), potentially contributing to disease pathogenesis, have been shown to be altered in several neurodegenerative diseases including AD (Lim et al., 2011; Hernandez-Guillamon et al., 2015; Chowdhury, 2016; Weekman and Wilcock, 2016), and ALS (Lim et al., 1996; Fang et al., 2010; Kaplan et al., 2014). Furthermore, increased CSF MMP-9 levels directly correlate with disease severity in HD patients (Connolly et al., 2016). To determine if these changes in MMP-2 and MMP-9 expression levels can be recapitulated in our iPSC-derived NSC lines, gelatin zymography was performed on cell lysates (Figure 4A) and conditioned media (Figure 4B) obtained from C116- and HD-NSCs. Although modest changes were observed in pro-MMP-2 levels, the zymogram demonstrated a marked decrease in active MMP-2 levels in HD-NSCs when compared to control C116-NSCs, a result further confirmed by immunofluorescence analysis (Figure 4C). Strong upregulation of MMP-2 expression was observed especially in association with apoptotic cells (punctate degenerating nuclei) (white arrowheads in Figure 4C), suggesting a role for MMP-2 in cellular apoptosis. Increased expression of pro-MMP-9 was observed in samples derived from HD-NSCs compared to those obtained from C116-NSCs (Figures 4A,B). Furthermore, immunofluorescence analysis again revealed strictly nuclear localization of MMP-9 in HD-NSCs, in contrast with the diffuse nuclear and cytoplasmic expression in C116-NSCs (Figure 4D).

### TIMP levels are modulated in HD

**Figure 5:**
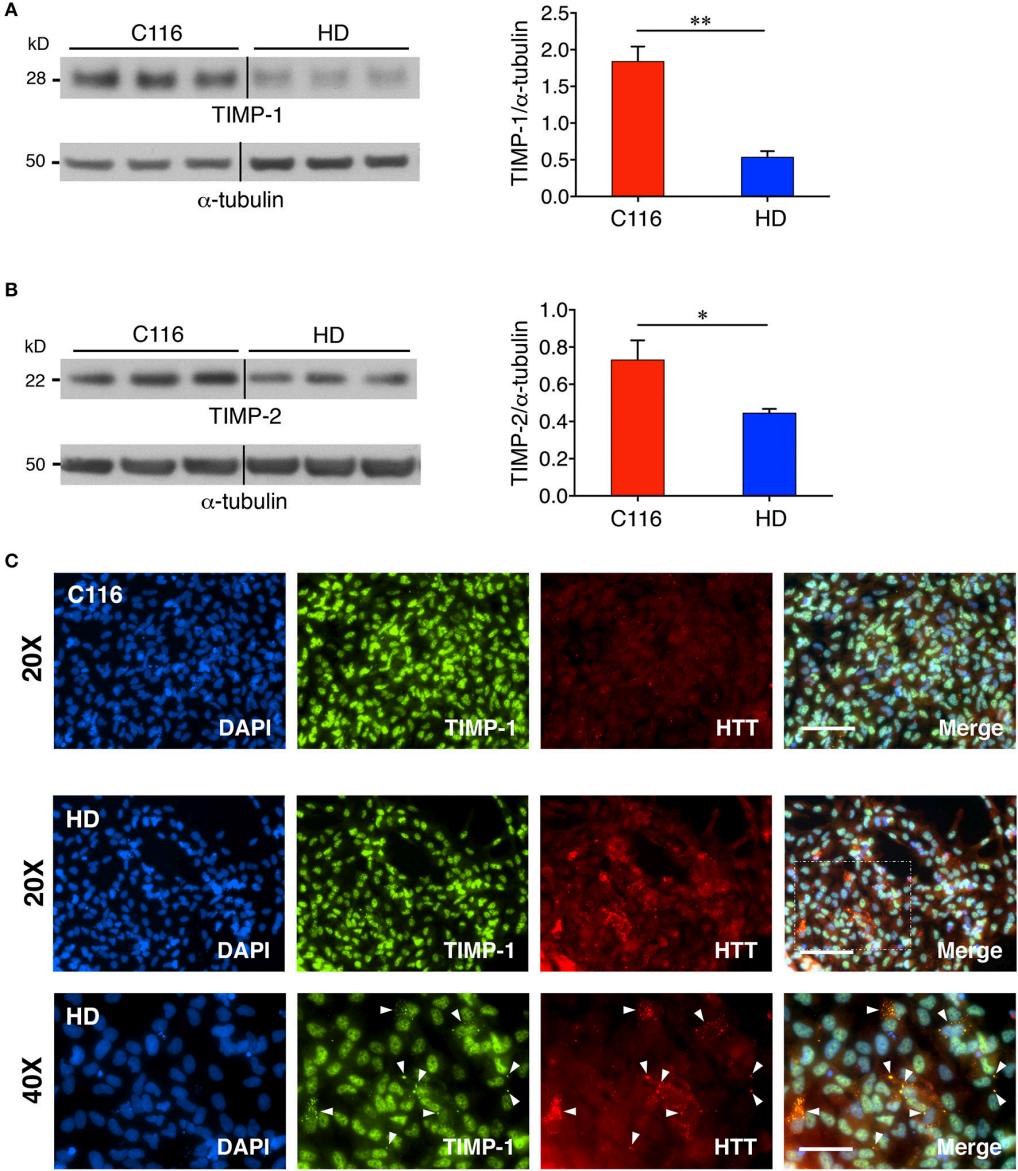
TIMP expression is downregulated in HD-NSCs. Western blot analysis of TIMP-1 **(A)** and TIMP-2 **(B)** expression reveals significantly decreased levels in HD-NSCs compared to C116-NSCs (*n* = 3 BR, ^*^p < 0.05; ^**^p < 0.01; *t*-test). Error bars represent *SD*. Immunofluorescence analysis using anti- TIMP-1 antibody **(C)** demonstrates nuclear localization of TIMP-1 and suggests a direct association between TIMP-1 and mHTT aggregates in HD-NSCs (40X magnified inset, white arrowheads). Scale Bars: 20X = 100 μm, 40X = 50 μm.

TIMPs are endogenous inhibitors of MMPs, and thus, altered MMP levels are often associated with concomitant changes in TIMP levels. Elevated levels of TIMPs have been reported in several neurodegenerative diseases, including PD, AD, HD, and ALS (Lorenzl et al., 2003a), where they have been ascribed roles in neuroprotection (Tan et al., 2003; Magnoni et al., 2007; Fujimoto et al., 2008; Tejima et al., 2009; Walker and Rosenberg, 2009; Kim et al., 2010; Ashutosh et al., 2012; Lee and Kim, 2014; Gibb et al., 2015), remyelination (Jiang et al., 2016) and maintaining CNS homeostasis (Gardner and Ghorpade, 2003). To determine if TIMP expression is modulated in HD, western blot analysis (and RT-PCR analysis) was performed on cell lysates prepared from C116- and HD-NSCs. Our results demonstrate a decreased expression of both TIMP-1 [Figure 5A (3.3-fold decrease), Supplementary Figure 1D (1.4-fold decrease)] and TIMP-2 [Figure 5B (1.6-fold decrease), Supplementary Figure 1E (1.2-fold decrease)] in HD-NSCs. The downregulation of these endogenous MMP inhibitors likely accounts for the unabated MMP activity in HD-NSCs. Immunocytochemistry analysis further revealed that nuclear TIMP-1 is found in association with nuclear HTT (Figure 5C) suggesting that TIMP-1 regulates MMP activity in the nucleus and potentially plays a role in inhibiting cellular apoptosis (Mannello and Gazzanelli, 2001). HD-NSCs readily form cytoplasmic perinuclear mHTT aggregates as detected by anti-huntingtin antibody (a.a. 115-129, MAB5490). Interestingly, there appears to be an interaction between TIMP-1 and mHTT aggregates in HD-NSCs (white arrowheads in Figure 5C, Supplementary Figure 2).

### TGF-β regulates TIMP-1 expression

**Figure 6:**
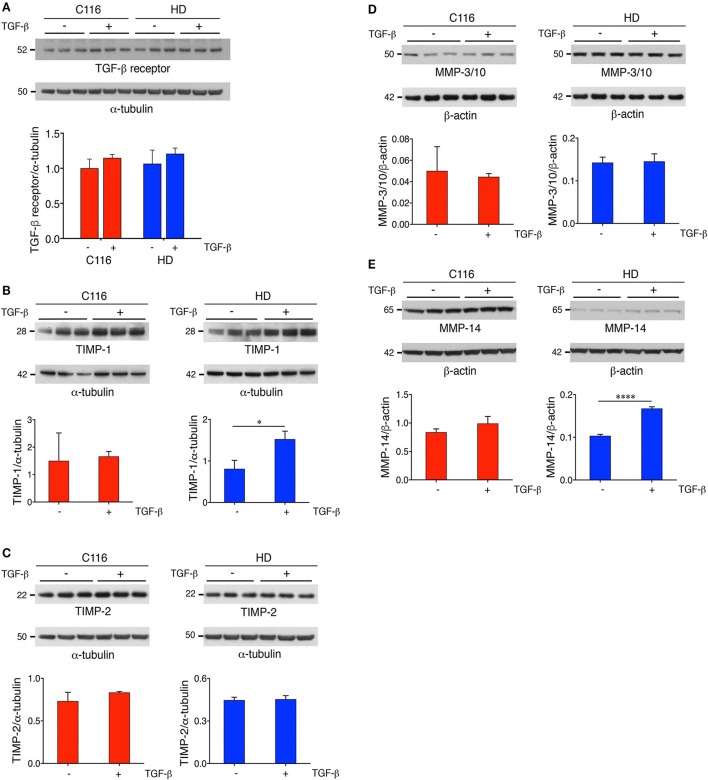
TGF-β treatment regulates TIMP-1 expression. C116- and HD-NSCs were treated with 10 ng/mL TGF-β for 24 h. **(A)** Immunoblot analysis demonstrates that both corrected C116- and HD-NSCs express the TGF-β receptor at equal levels, and that exogenous TGF-β administration does not modulate expression of its cognate receptor (ANOVA with Tukey's multiple comparison test). Western blot analysis reveals that TGF-β treatment resulted in increased expression of TIMP-1 in HD-NSCs **(B)**, but not in C116-NSCs. No changes are observed in the expression levels of TIMP-2 **(C)** and MMP-3/10 **(D)** in response to TGF-β, however, a very modest increase is observed in MMP-14 expression in HD-NSCs **(E)** (^*^p < 0.05; ^****^p < 0.0001; *t*-test). Error bars represent *SD*.

**Figure 7:**
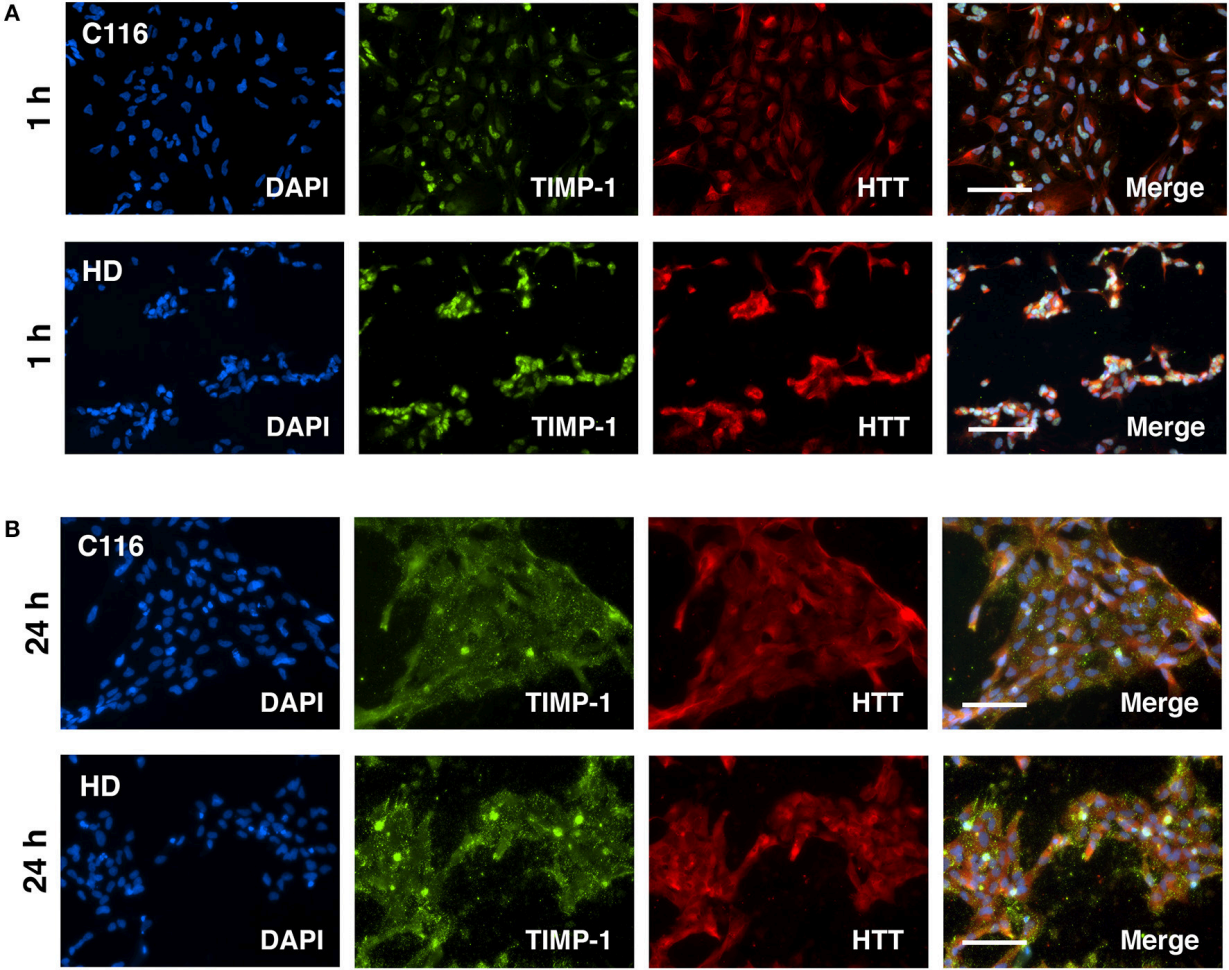
Altered localization of TIMP-1 in response to TGF-β treatment as a function of time. C116- and HD-NSCs were treated with 20 ng/mL TGF-β. **(A)** Immunofluorescence analysis shows robust induction of TIMP-1 levels in HD-NSCs. Strong nuclear expression of TIMP-1 is observed in both C116- and HD-NSCs. However, the expression becomes predominantly cytoplasmic after 24 h of TGF-β treatment **(B)** across both genotypes. Scale Bars: 100 μm.

Our previous studies on differential gene expression and Ingenuity Pathway Analysis (IPA) of HD-NSCs and corrected C116-NSCs highlighted TGF-β as the top dysregulated pathway (Ring et al., 2015). HD-NSCs expressed significantly higher levels of TGF-β in all its forms (precursor, monomer, and dimer) compared to C116-NSCs. Exogenous addition of TGF-β conferred neuroprotection in HD-NSCs as indicated by amelioration of elevated caspase-3/7 activity, a direct measure of cellular apoptosis, and by rescuing mitochondrial deficits. Altered levels of TGF-β have been reported in HD mouse models and peripheral blood of HD patients as well (Battaglia et al., 2011). Although very little is known about TGF-β-mediated regulation of MMP/TIMP activity, recent studies have shown that TGF-β modulates the homeostasis between MMPs and TIMPs through putative signaling pathways (Hall et al., 2003; Gomes et al., 2012; Kwak, 2013). To determine if TGF-β regulates MMP and TIMP expression in C116- and HD-NSCs, cells were treated with 10 ng/mL TGF-β for 24 h. Western blot analysis revealed that both cell lines expressed the TGF-β receptor at basal conditions (Figure 6A), and that TGF-β treatment did not alter the expression levels of its cognate receptor. Interestingly, treatment with TGF-β resulted in significantly increased expression of TIMP-1 in HD-NSCs, but not in C116-NSCs (Figure 6B). No significant change in expression of TIMP-2 (Figure 6C), or MMP-3/10 (Figure 6D) was observed. A very modest increase in MMP-14 expression in HD-NSCs (Figure 6E) was observed in response to TGF-β. To further confirm these finding, C116- and HD-NSCs were treated with a higher dose of 20 ng/mL TGF-β for 24 h. A robust upregulation of TIMP-1 expression was observed not only in HD-NSCs, but also in C116-NSCs (Supplementary Figure 3A). These results together indicate that corrected C116-NSCs have a higher threshold for TIMP-1 induction. Consistent with previous findings, very little modulation of TIMP-2 expression was observed at the higher TGF-β dose in across both genotypes (Supplementary Figure 3B). These data suggest that one of the possible mechanisms by which TGF-β exerts its neuroprotective effects is through the upregulation of TIMP-1 expression. Finally, altered localization of TIMP-1 expression was observed as a function of time in response to treatment with 20 ng/mL TGF-β, with TIMP-1 expression shifting from being strictly nuclear at 1 h post-treatment (Figure 7A), to completely cytoplasmic at 24 h post-treatment (Figure 7B), as revealed by immunofluorescence analysis.

## Discussion

Our study successfully demonstrates altered expression of MMPs and TIMPs across the corrected C116- and HD-NSC lines. Previous studies in our lab demonstrated that MMP-10 colocalized with mHTT aggregates in discrete punctate structures and that MMP-10 directly cleaves mHTT into N-terminal toxic fragments (Miller et al., 2010). Since MMP-10 shares a very high degree of homology with MMP-3 (86% identical catalytic domains) (Bertini et al., 2004), MMP-3 may very well be implicated in contributing to mHTT cleavage and toxicity as well. Our results showing a significant increase in MMP-3/10 activity in HD-NSCs suggests that mHTT toxicity occurs in early stages of neuronal cell development in HD. A study has shown that neurons that are under cellular stress release the active form of MMP-3, which in turn triggers microglial activation and production of pro-inflammatory cytokines including Tumor necrosis factor α (TNF-α), interleukin-6 (IL-6) and IL-1 (Kim et al., 2005), thereby contributing to the neuroinflammatory response in the HD brain (Bjorkqvist et al., 2008), and worsening of disease (Connolly et al., 2016). Furthermore, nuclear presence of MMP-3 (as shown in Figure 2B) was found to be associated with caspase-3 mediated cellular apoptosis (Mannello et al., 2005; Si-Tayeb et al., 2006; Choi et al., 2008), thus contributing to neuroinflammation and neuronal damage (Kim and Hwang, 2011). These studies put together indicate that specific inhibition of MMP-3 and MMP-10 activity may prove to be of therapeutic value in treating HD.

Contrary to our previous findings that MMP-14 levels were elevated in Hdh^111Q/111Q^ (Miller et al., 2010), diminished expression of MMP-14 was observed in HD-NSCs. This can be attributed to change in steady-state levels of MMP-14 during activation and processing of this enzyme or to difference in cell type or species since previous studies involved immortalized mouse striatal cell lines vs. human NSCs in this study. In fact, elevated MMP-14 levels in HD-NSCs may be beneficial as indicated by a study showing that elevated MMP-14 levels found in association with reactive astrocytes surrounding amyloid deposits exhibited Aβ-degrading activity *in vitro* (Liao and Van Nostrand, 2010). Thus, boosting MMP-14 levels may help facilitate Aβ clearance and be of therapeutic benefit in AD. Similarly, it would be interesting to explore whether MMP-14 directly interacts with mHTT containing aggregates as well, thereby facilitating mHTT clearance and diminishing associated toxicity in HD.

**Figure 8:**
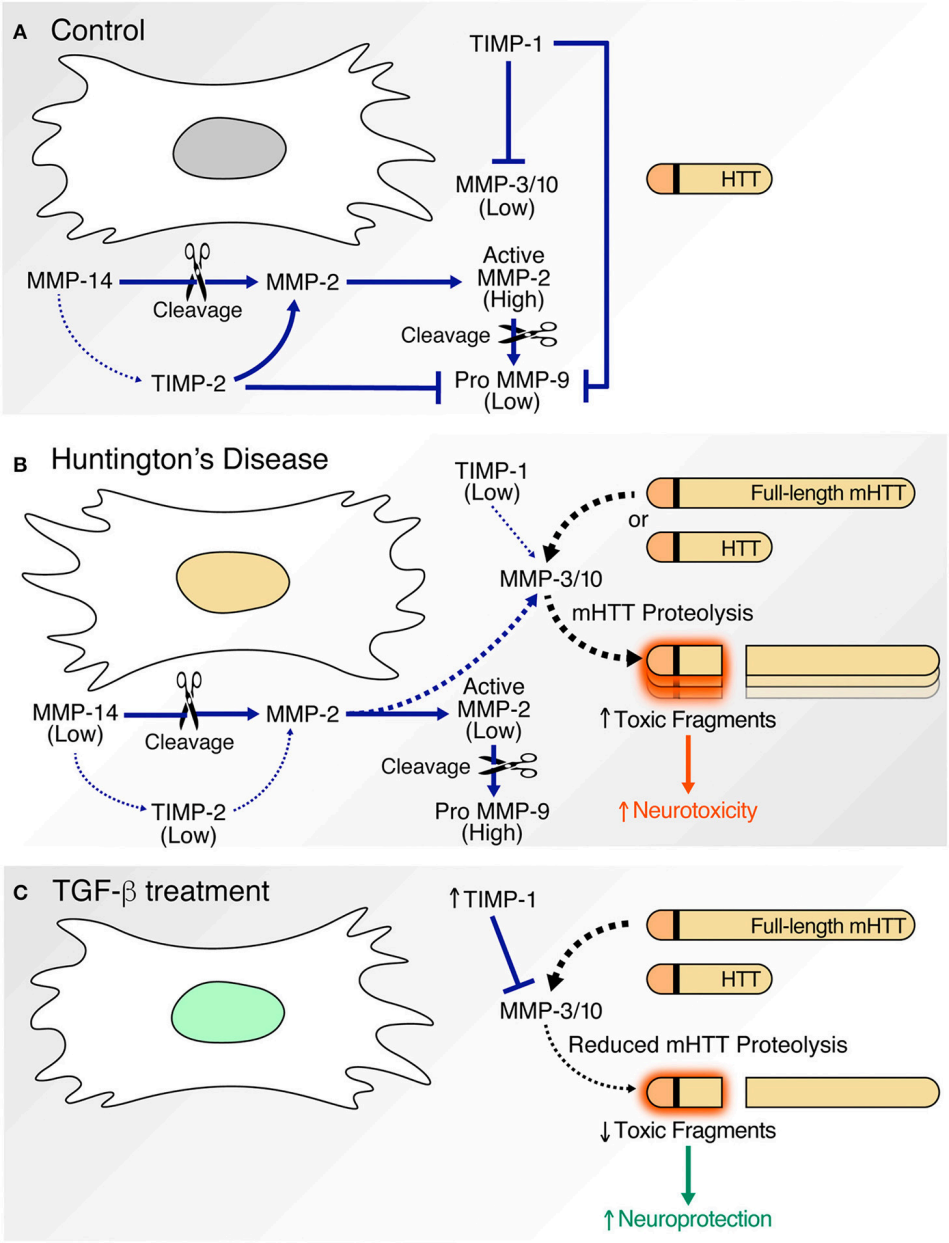
Proposed model. In comparison to corrected C116-NSCs **(A)**, HD-NSCs express altered levels of MMPs and TIMPs **(B)**. **(A)** Under basal conditions, MMP-3/10 activity is kept in check by TIMP-1. MMP-14, MMP-2, and TIMP-2 form a trimolecular complex that facilitates activation of MMP-2, which in turn induces MMP-9. MMP-9 activity is greatly inhibited by TIMPs. **(B)** Decreased expression of TIMPs in HD-NSCs result in unregulated MMP activity as indicated by elevated MMP-3/10 expression. MMP-3/10 directly cleaves full-length mutant HTT (mHTT) leading to the production of toxic N-terminal polyQ fragments, consequently contributing to increased cell death in HD-NSCs. Decreased expression of TIMP-2 and MMP-14 result in reduced activation of MMP-2. Increased MMP-9 activity is observed possibly as a result of decreased TIMP expression. **(C)** TGF-β treatment upregulates TIMP-1 expression, which in turn inhibits MMP-3/10 activity. This inhibition prevents MMP-3/10-mediated cleavage of full-length mHTT, thereby decreasing the production of toxic N-terminal fragments and amelioration of cell death. Thus, TGF-β conferred neuroprotection is likely mediated through increased expression of TIMP-1.

Our gelatin zymography studies clearly demonstrate altered activation of MMP-2 and -9 in HD-NSCs. While increased expression of MMP-9 was observed in HD-NSCs, activation of MMP-2 was diminished. Since a ternary complex of pro-MMP-2, TIMP-2, and MMP-14 is required for activation of MMP-2 (Nagase, 1998; Itoh and Seiki, 2004) (Figure 8), decreased expression of MMP-14 and TIMP-2 in HD-NSCs possibly accounts for decreased levels of active MMP-2.

Our immunofluorescence analysis indicated that increased nuclear MMP-2 and -9 expression is associated with degenerating nuclei in apoptotic cells. Much like caspase-3 activity, intranuclear MMP-2 and -9 have been shown to have pro-apoptotic functions in neurons by hindering DNA strand break repair through inactivation of poly-ADP-ribose-polymerase-1 (PARP-1), a DNA repair enzyme (Mannello et al., 2005; Hill et al., 2012). Intranuclear MMP activity in neurons has also been associated with neuroinflammation (Kimura-Ohba and Yang, 2016).

The biology of the MMP-2 and -9 is truly complex. Contrary to the above studies, these gelatinases were also shown to have beneficial roles in AD. When incubated with Aβ40 and Aβ42, MMP-2, and -9 sequentially degraded them into highly soluble, non-toxic truncated fragments, thereby facilitating Aβ clearance (Hernandez-Guillamon et al., 2015). Thus, it would be interesting to study potential interactions of expanded mHTT with recombinant MMP-2 and MMP-9.

MMP-2 and -9 have been widely implicated in pathogenesis of several neurodegenerative diseases including AD, HD, and ALS. Decreased levels of plasma MMP-2 (Lim et al., 2011) and elevated levels of MMP-9 in post-mortem cortical tissue (Bruno et al., 2009), CSF samples (Adair et al., 2004), and plasma samples (Lorenzl et al., 2003b) of AD patients have been reported, and shown to bear direct correlation with worsening Mini-Mental State Examination (MMSE) scores (Bruno et al., 2009; Lim et al., 2011). These studies clearly underscore the clinical utility of these MMPs as valuable prognostic biomarkers of neurodegenerative disease progression. Furthermore, MMP-9 inhibition was reported to improve Aβ-mediated cognitive impairment and neurotoxicity in mice (Mizoguchi et al., 2009). On similar lines, studies by Kaplan et al. demonstrated that MMP-9 is a prospective marker for motor neuron loss in late-stage ALS, and that reduction of MMP-9 function through gene ablation, viral gene therapy, and pharmacological inhibition significantly delayed muscle denervation (Kaplan et al., 2014). Additionally, a positive correlation was found between significantly increased MMP-9 CSF levels and disease severity in HD (Connolly et al., 2016). Thus, MMP-9 appears to be a good candidate therapeutic target for treatment of several neurodegenerative disorders including HD.

Western blot analysis further demonstrated that both TIMP-1 and TIMP-2 expression was downregulated in HD-NSCs compared to C116-NSCs, thereby directly accounting for the altered MMP levels in HD-NSCs. However, TIMPs are pleiotropic and also serve MMP-independent functions in the central nervous system (CNS) (Stetler-Stevenson, 2008; Moore and Crocker, 2012). Both TIMP-1 and TIMP-2 are anti-apoptotic (Mannello and Gazzanelli, 2001; Brew and Nagase, 2010) and have direct roles in conferring neuroprotection (Kim et al., 2010; Ashutosh et al., 2012). Nuclear localization of TIMPs, especially TIMP-1, has been previously reported (Li et al., 1995; Zhao et al., 1998), inviting the speculation that they have important roles in nuclear functions such as replication and transcription (Mannello and Medda, 2012). Furthermore, association of TIMP-1 with mHTT aggregates in discrete punctate structures as revealed by immunofluorescence analysis suggests a direct physical interaction of TIMP-1 with mHTT. Presence of TIMP-1 in these mHTT aggregates possibly serves to directly inhibit MMP-10 activity, which has been reported to strongly colocalize with mHTT aggregates as well (Miller et al., 2010).

Studies by Moore et al. show that compact myelin formation is significantly delayed in TIMP-1 knockout mice, and that astrocytic TIMP-1 promotes oligodendrocyte differentiation and enhances CNS myelination (Moore et al., 2011). Furthermore, transplantation of immature iPSC-derived astrocytes or administration of their conditioned medium promoted recovery following brain injury in a TIMP-1 dependent manner (Jiang et al., 2016). TIMP-1 overexpression also ameliorates MMP-9 mediated blood-brain barrier leakage in models of stroke, traumatic brain injury (Tejima et al., 2009) and cerebral ischemia (Fujimoto et al., 2008). Thus, astrocytic TIMP-1 plays an important role in CNS homeostasis and disease and may have significant therapeutic relevance (Gardner and Ghorpade, 2003).

TIMP-2 plays an equally important role in the CNS. TIMP-2 is expressed in spinal motor neurons, and is necessary for development and maintenance of the neuromuscular junction, as indicated by the presence of motor deficits in TIMP-2 deficient mice (Jaworski et al., 2006). TIMP-2 also has a potent anti-inflammatory role. Overexpression of TIMP-2 in LPS-stimulated microglia inhibited the production of reactive oxygen species and proinflammatory cytokines, including TNF-α and IL-1β, while simultaneously increasing production of anti-inflammatory IL-10 (Lee and Kim, 2014). TNF-α and IL-6 were found to be significantly elevated in postmortem HD patient samples and in mouse models of HD (Bjorkqvist et al., 2008), and thus, boosting TIMP-2 levels in the HD brain could attenuate the neuroinflammatory response, thus providing therapeutic benefit.

As with MMPs, TIMPs can also be valuable biomarkers for neurodegenerative diseases (Lorenzl et al., 2003a). Studies by Mroczko et al. showed that simultaneous examination of MMP-2, -9, and TIMP-1 in the CSF allowed for differential diagnosis between AD and other types of dementia (Mroczko et al., 2013). Elevated CSF levels of TIMP-1 and TIMP-2 in HD patients, which probably represent a compensatory attempt at controlling unabated MMP activity, could also be potential biomarkers for HD (Lorenzl et al., 2003a; Connolly et al., 2016).

A proposed mechanism for our findings illustrates the contribution of altered MMP and TIMP expression to HTT proteolysis and neuronal cell death (Figure 8). Under basal conditions (Figure 8A), MMP activity is tightly regulated by TIMPs. MMP-2, MMP-14, and TIMP-2 form a ternary complex (Nagase, 1998; Itoh and Seiki, 2004). MMP-2 zymogen is proteolytically processed into its active form by MMP-14, and MMP-2 further activates MMP-9. TIMPs inhibit the activity of MMP-3/10 (Batra et al., 2012), MMP-2, and MMP-9 (Fujimoto et al., 2008; Tejima et al., 2009). In HD (Figure 8B), decreased TIMP-2 levels result in reduced MMP-2 activation. MMP-2 further activates MMP-9 and potentially also activates MMP-3/10. Reduced TIMP levels in HD-NSCs simultaneously contribute to unabated MMP-3/10 and MMP-9 activity. Previous work in the lab has shown that MMP-3/10 directly cleaves full-length mHTT into smaller N-terminal fragments which associate with increased neurotoxicity. Thus, elevated CSF levels of TIMPs (TIMP-1 and TIMP-2) in neurodegenerative diseases, including HD (Lorenzl et al., 2003a), represent a compensatory attempt at regulating already elevated and uncontrolled MMP activity. Exogenous administration of TGF-β (Figure 8C) increases TIMP-1 expression in HD-NSCs, thereby inhibiting MMP-3/10 expression and concomitant proteolysis of mHTT into toxic fragments. In conclusion, upregulation of TIMP-1 expression represents a potential mechanism for TGF-β conferred-neuroprotection in HD (Ring et al., 2015). Thus, boosting TGF-β signaling could potentially offer therapeutic benefit in HD. In fact, a recent study demonstrated that deficiency in neuronal TGF-β signaling promoted PD-related pathologies and motor deficits (Tesseur et al., 2017). Increasing TGF-β signaling in the substantia nigra through adeno-associated virus expressing a constitutively active type I receptor significantly reduced MPTP-induced dopaminergic neurodegeneration and motor deficits, and thus, represents a potential therapy for PD. This study also indicates that boosting TGF-β signaling through constitutively activating TGF-β receptor in a cell-type specific manner is more beneficial compared to *in vivo* delivery of TGF-β ligands, which has yielded contradictory results (Krieglstein and Unsicker, 1994; Poulsen et al., 1994; Krieglstein et al., 1995; Sanchez-Capelo et al., 2003), possibly due to global activation of TGF-β signaling. Thus, we speculate that augmenting TGF-β signaling specifically in the striatum potentially represents a feasible strategy for treatment of HD as well.

Our study also indicates that MMP inhibition can provide therapeutic benefit in HD. However, multiple MMPs are expressed in all cell types throughout the body, with some having crucial multifaceted roles in normal physiology (Brkic et al., 2015), thereby emphasizing the need for developing very specific MMP inhibitors. This has been exemplified by drug discovery efforts in the cancer field showing that more than 50 MMP inhibitors investigated have failed in clinical trials, owing to lack of inhibitor specificity (Vandenbroucke and Libert, 2014). For instance, administration of broad-spectrum MMP inhibitors resulted in musculoskeletal syndrome manifesting as stiffening of joints, hypothesized to be due to inhibition of MMPs crucial to maintaining connective tissue homeostasis, namely MMP-1 and MMP-14 (Becker et al., 2010). In the light of these findings, efforts are now focused on developing highly specific MMP inhibitors that spare off-target isozymes. These studies underscore the need to develop targeted MMP inhibition therapies for treatment of HD.

In conclusion, elucidating the mechanism of action of MMPs and TIMPs, their interactions with mHTT, and their regulation by other cytokines or neurotrophic factors will shed light on their contribution to HD pathology, and help develop novel therapies for treatment of HD. Findings of this study will be broadly applicable to other neurodegenerative diseases and normal aging as well.

## Author contributions

LME: designed the project, organized the entire research, and provided the financial support; AE, SN, KM, and KR: conducted the experiments; SN and LME: performed data analysis and interpretation; AE, SN, and LME: wrote the manuscript; SN and LME: revised the manuscript; LME approved the final manuscript; All authors discussed the results and reviewed the manuscript.

### Conflict of interest statement

The authors declare that the research was conducted in the absence of any commercial or financial relationships that could be construed as a potential conflict of interest.

## References

[B1] Adair J. C. Charlie J. Dencoff J. E. Kaye J. A. Quinn J. F. Camicioli R. M.. (2004). Measurement of gelatinase B (MMP-9) in the cerebrospinal fluid of patients with vascular dementia and alzheimer disease. Stroke 35, e159–e162. 10.1161/01.STR.0000127420.10990.76 15105518

[B2] An M. C. Zhang N. Scott G. Montoro D. Wittkop T. Mooney S.. (2012). Genetic correction of Huntington's disease phenotypes in induced pluripotent stem cells. Cell Stem Cell 11, 253–263. 10.1016/j.stem.2012.04.026 22748967 PMC3608272

[B3] Ashutosh C. C. Borgmann K. Brew K. Ghorpade A. (2012). Tissue inhibitor of metalloproteinases-1 protects human neurons from staurosporine and HIV-1-induced apoptosis: mechanisms and relevance to HIV-1-associated dementia. Cell Death Dis. 3:e332. 10.1038/cddis.2012.54 22739984 PMC3388228

[B4] Batra J. Robinson J. Soares A. S. Fields A. P. Radisky D. C. Radisky E. S. (2012). Matrix metalloproteinase-10 (MMP-10) interaction with tissue inhibitors of metalloproteinases TIMP-1 and TIMP-2: binding studies and crystal structure. J. Biol. Chem. 287, 15935–15946. 10.1074/jbc.M112.341156 22427646 PMC3346077

[B5] Battaglia G. Cannella M. Riozzi B. Orobello S. Maat-Schieman M. L. Aronica E.. (2011). Early defect of transforming growth factor beta1 formation in Huntington's disease. J. Cell. Mol. Med. 15, 555–571. 10.1111/j.1582-4934.2010.01011.x 20082658 PMC3922377

[B6] Becker D. P. Barta T. E. Bedell L. J. Boehm T. L. Bond B. R. Carroll J.. (2010). Orally active MMP-1 sparing alpha-tetrahydropyranyl and alpha-piperidinyl sulfone matrix metalloproteinase (MMP) inhibitors with efficacy in cancer, arthritis, and cardiovascular disease. J. Med. Chem. 53, 6653–6680. 10.1021/jm100669j 20726512

[B7] Bertini I. Calderone V. Fragai M. Luchinat C. Mangani S. Terni B. (2004). Crystal structure of the catalytic domain of human matrix metalloproteinase 10. J. Mol. Biol. 336, 707–716. 10.1016/j.jmb.2003.12.033 15095982

[B8] Bjorkqvist M. Wild E. J. Thiele J. Silvestroni A. Andre R. Lahiri N.. (2008). A novel pathogenic pathway of immune activation detectable before clinical onset in Huntington's disease. J. Exp. Med. 205, 1869–1877. 10.1084/jem.20080178 18625748 PMC2525598

[B9] Brew K. Nagase H. (2010). The tissue inhibitors of metalloproteinases (TIMPs): an ancient family with structural and functional diversity. Biochim. Biophys. Acta 1803, 55–71. 10.1016/j.bbamcr.2010.01.003 20080133 PMC2853873

[B10] Brkic M. Balusu S. Libert C. Vandenbroucke R. E. (2015). Friends or foes: matrix metalloproteinases and their multifaceted roles in neurodegenerative diseases. Mediators Inflamm. 2015:620581. 10.1155/2015/620581 26538832 PMC4619970

[B11] Bruno M. A. Mufson E. J. Wuu J. Cuello A. C. (2009). Increased matrix metalloproteinase 9 activity in mild cognitive impairment. J. Neuropathol. Exp. Neurol. 68, 1309–1318. 10.1097/NEN.0b013e3181c22569 19915485 PMC2810197

[B12] Choi D. H. Kim E. M. Son H. J. Joh T. H. Kim Y. S. Kim D.. (2008). A novel intracellular role of matrix metalloproteinase-3 during apoptosis of dopaminergic cells. J. Neurochem. 106, 405–415. 10.1111/j.1471-4159.2008.05399.x 18397366

[B13] Chowdhury A, (2016). A diverse role of MMP-2 and MMP-9 in the onset of alzheimer disease and cancer. Austin Neurol. Neurosci. 1, 1011–1015.

[B14] Connolly C. Magnusson-Lind A. Lu G. Wagner P. K. Southwell A. L. Hayden M. R.. (2016). Enhanced immune response to MMP3 stimulation in microglia expressing mutant huntingtin. Neuroscience 325, 74–88. 10.1016/j.neuroscience.2016.03.031 27033979

[B15] Crocker S. J. Pagenstecher A. Campbell I. L. (2004). The TIMPs tango with MMPs and more in the central nervous system. J. Neurosci. Res. 75, 1–11. 10.1002/jnr.10836 14689443

[B16] Fang L. Teuchert M. Huber-Abel F. Schattauer D. Hendrich C. Dorst J.. (2010). MMP-2 and MMP-9 are elevated in spinal cord and skin in a mouse model of ALS. J. Neurol. Sci. 294, 51–56. 10.1016/j.jns.2010.04.005 20441996

[B17] Fujimoto M. Takagi Y. Aoki T. Hayase M. Marumo T. Gomi M.. (2008). Tissue inhibitor of metalloproteinases protect blood-brain barrier disruption in focal cerebral ischemia. J. Cereb. Blood Flow Metab. 28, 1674–1685. 10.1038/jcbfm.2008.59 18560439

[B18] Gafni J. Hermel E. Young J. E. Wellington C. L. Hayden M. R. Ellerby L. M. (2004). Inhibition of calpain cleavage of huntingtin reduces toxicity: accumulation of calpain/caspase fragments in the nucleus. J. Biol. Chem. 279, 20211–20220. 10.1074/jbc.M401267200 14981075

[B19] Gardner J. Ghorpade A. (2003). Tissue inhibitor of metalloproteinase (TIMP)-1: the TIMPed balance of matrix metalloproteinases in the central nervous system. J. Neurosci. Res. 74, 801–806. 10.1002/jnr.10835 14648584 PMC3857704

[B20] Gibb S. L. Zhao Y. Potter D. Hylin M. J. Bruhn R. Baimukanova G.. (2015). TIMP3 Attenuates the loss of neural stem cells, mature neurons and neurocognitive dysfunction in traumatic brain injury. Stem Cells 33, 3530–3544. 10.1002/stem.2189 26299440

[B21] Gomes L. R. Terra L. F. Wailemann R. A. Labriola L. Sogayar M. C. (2012). TGF-beta1 modulates the homeostasis between MMPs and MMP inhibitors through p38 MAPK and ERK1/2 in highly invasive breast cancer cells. BMC Cancer 12:26. 10.1186/1471-2407-12-26 22260435 PMC3277461

[B22] Graham R. K. Deng Y. Slow E. J. Haigh B. Bissada N. Lu G.. (2006). Cleavage at the caspase-6 site is required for neuronal dysfunction and degeneration due to mutant huntingtin. Cell 125, 1179–1191. 10.1016/j.cell.2006.04.026 16777606

[B23] Hall M. C. Young D. A. Waters J. G. Rowan A. D. Chantry A. Edwards D. R.. (2003). The comparative role of activator protein 1 and smad factors in the regulation of Timp-1 and MMP-1 gene expression by transforming growth factor-beta 1. J. Biol. Chem. 278, 10304–10313. 10.1074/jbc.M212334200 12525489

[B24] He X. Zhang L. Yao X. Hu J. Yu L. Jia H.. (2013). Association studies of MMP-9 in Parkinson's disease and amyotrophic lateral sclerosis. PLoS ONE 8:e73777. 10.1371/journal.pone.0073777 24040066 PMC3767588

[B25] Hernandez-Guillamon M. Mawhirt S. Blais S. Montaner J. Neubert T. A. Rostagno A.. (2015). Sequential amyloid-beta degradation by the matrix metalloproteases MMP-2 and MMP-9. J. Biol. Chem. 290, 15078–15091. 10.1074/jbc.M114.610931 25897080 PMC4463451

[B26] Hill J. W. Poddar R. Thompson J. F. Rosenberg G. A. Yang Y. (2012). Intranuclear matrix metalloproteinases promote DNA damage and apoptosis induced by oxygen-glucose deprivation in neurons. Neuroscience 220, 277–290. 10.1016/j.neuroscience.2012.06.019 22710064 PMC4546359

[B27] Itoh Y. Seiki M. (2004). MT1-MMP: an enzyme with multidimensional regulation. Trends Biochem. Sci. 29, 285–289. 10.1016/j.tibs.2004.04.001 15276180

[B28] Jaworski D. M. Soloway P. Caterina J. Falls W. A. (2006). Tissue inhibitor of metalloproteinase-2(TIMP-2)-deficient mice display motor deficits. J. Neurobiol. 66, 82–94. 10.1002/neu.20205 16216006 PMC1440718

[B29] Jiang P. Chen C. Liu X. B. Pleasure D. E. Liu Y. Deng W. (2016). Human iPSC-derived immature astroglia promote oligodendrogenesis by increasing TIMP-1 secretion. Cell Rep. 15, 1303–1315. 10.1016/j.celrep.2016.04.011 27134175 PMC4864503

[B30] Kaplan A. Spiller K. J. Towne C. Kanning K. C. Choe G. T. Geber A.. (2014). Neuronal matrix metalloproteinase-9 is a determinant of selective neurodegeneration. Neuron 81, 333–348. 10.1016/j.neuron.2013.12.009 24462097 PMC6015650

[B31] Kapoor C. Vaidya S. Wadhwan V. Hitesh Kaur G. Pathak A. (2016). Seesaw of matrix metalloproteinases (MMPs). J. Cancer Res. Ther. 12, 28–35. 10.4103/0973-1482.157337 27072206

[B32] Kim E. M. Hwang O. (2011). Role of matrix metalloproteinase-3 in neurodegeneration. J. Neurochem. 116, 22–32. 10.1111/j.1471-4159.2010.07082.x 21044079

[B33] Kim S. Y. Woo M. S. Park J. S. Hyun J. W. Kim Y. S. Kim H. S. (2010). The neuroprotective role of tissue inhibitor of metalloproteinase-2 in MPP+- or 6-OHDA-treated SK-N-BE(2)C and SH-SY5Y human neuroblastoma cells. Neurosci. Lett. 468, 136–140. 10.1016/j.neulet.2009.10.084 19883732

[B34] Kim Y. S. Kim S. S. Cho J. J. Choi D. H. Hwang O. Shin D. H.. (2005). Matrix metalloproteinase-3: a novel signaling proteinase from apoptotic neuronal cells that activates microglia. J. Neurosci. 25, 3701–3711. 10.1523/JNEUROSCI.4346-04.2005 15814801 PMC6725382

[B35] Kimura-Ohba S. Yang Y. (2016). Oxidative DNA damage mediated by intranuclear MMP activity Is associated with neuronal apoptosis in ischemic stroke. Oxid. Med. Cell. Longev. 2016:6927328. 10.1155/2016/6927328 26925194 PMC4748094

[B36] Krieglstein K. Suter-Crazzolara C. Fischer W. H. Unsicker K. (1995). TGF-beta superfamily members promote survival of midbrain dopaminergic neurons and protect them against MPP+ toxicity. EMBO J. 14, 736–742. 7882977 10.1002/j.1460-2075.1995.tb07052.x PMC398139

[B37] Krieglstein K. Unsicker K. (1994). Transforming growth factor-beta promotes survival of midbrain dopaminergic neurons and protects them against N-methyl-4-phenylpyridinium ion toxicity. Neuroscience 63, 1189–1196. 10.1016/0306-4522(94)90583-5 7700516

[B38] Kwak H. B. (2013). Aging, exercise, and extracellular matrix in the heart. J. Exerc. Rehabil. 9, 338–347. 10.12965/jer.130049 24278882 PMC3836529

[B39] Lee E. J. Kim H. S. (2014). The anti-inflammatory role of tissue inhibitor of metalloproteinase-2 in lipopolysaccharide-stimulated microglia. J. Neuroinflam. 11, 116. 10.1186/1742-2094-11-116 24970341 PMC4091675

[B40] Li H. Nishio K. Yamashita K. Hayakawa T. Hoshino T. (1995). Cell cycle-dependent localization of tissue inhibitor of metalloproteinases-1 immunoreactivity in cultured human gingival fibroblasts. Nagoya J. Med. Sci. 58, 133–142. 8725497

[B41] Liao M. C. Van Nostrand W. E. (2010). Degradation of soluble and fibrillar amyloid beta-protein by matrix metalloproteinase (MT1-MMP) *in vitro*. Biochemistry 49, 1127–1136. 10.1021/bi901994d 20050683 PMC2819544

[B42] Lim G. P. Backstrom J. R. Cullen M. J. Miller C. A. Atkinson R. D. Tokes Z. A. (1996). Matrix metalloproteinases in the neocortex and spinal cord of amyotrophic lateral sclerosis patients. J. Neurochem. 67, 251–259. 10.1046/j.1471-4159.1996.67010251.x 8666998

[B43] Lim N. K. Villemagne V. L. Soon C. P. Laughton K. M. Rowe C. C. McLean C. A.. (2011). Investigation of matrix metalloproteinases, MMP-2 and MMP-9, in plasma reveals a decrease of MMP-2 in Alzheimer's disease. J. Alzheimers. Dis. 26, 779–786. 10.3233/JAD-2011-101974. 21694463

[B44] Lorenzl S. Albers D. S. LeWitt P. A. Chirichigno J. W. Hilgenberg S. L. Cudkowicz M. E.. (2003a). Tissue inhibitors of matrix metalloproteinases are elevated in cerebrospinal fluid of neurodegenerative diseases. J. Neurol. Sci. 207, 71–76. 10.1016/S0022-510X(02)00398-2 12614934

[B45] Lorenzl S. Albers D. S. Narr S. Chirichigno J. Beal M. F. (2002). Expression of MMP-2, MMP-9, and MMP-1 and their endogenous counterregulators TIMP-1 and TIMP-2 in postmortem brain tissue of parkinson's disease. Exp. Neurol. 178, 13–20. 10.1006/exnr.2002.8019 12460604

[B46] Lorenzl S. Albers D. S. Relkin N. Ngyuen T. Hilgenberg S. L. Chirichigno J.. (2003b). Increased plasma levels of matrix metalloproteinase-9 in patients with Alzheimer's disease. Neurochem. Int. 43, 191–196. 10.1016/S0197-0186(03)00004-4 12689599

[B47] Magnoni S. Baker A. Thomson S. Jordan G. George S. J. McColl B. W.. (2007). Neuroprotective effect of adenoviral-mediated gene transfer of TIMP-1 and−2 in ischemic brain injury. Gene. Ther. 14, 621–625. 10.1038/sj.gt.3302894 17235293

[B48] Malemud C. J. (2006). Matrix metalloproteinases (MMPs) in health and disease: an overview. Front. Biosci. 11, 1696–1701. 10.2741/1915 16368548

[B49] Mannello F. Gazzanelli G. (2001). Tissue inhibitors of metalloproteinases and programmed cell death: conundrums, controversies and potential implications. Apoptosis 6, 479–482. 10.1023/A:1012493808790 11595838

[B50] Mannello F. Luchetti F. Falcieri E. Papa S. (2005). Multiple roles of matrix metalloproteinases during apoptosis. Apoptosis 10, 19–24. 10.1007/s10495-005-6058-7 15711919

[B51] Mannello F. Medda V. (2012). Nuclear localization of matrix metalloproteinases. Prog. Histochem. Cytochem. 47, 27–58. 10.1016/j.proghi.2011.12.002 22226510

[B52] Mende-Mueller L. M. Toneff T. Hwang S. R. Chesselet M. F. Hook V. Y. (2001). Tissue-specific proteolysis of Huntingtin (htt) in human brain: evidence of enhanced levels of N- and C-terminal htt fragments in Huntington's disease striatum. J. Neurosci. 21, 1830–1837. 11245667 10.1523/JNEUROSCI.21-06-01830.2001 PMC6762596

[B53] Miller J. P. Holcomb J. Al-Ramahi I. de Haro M. Gafni J. Zhang N.. (2010). Matrix metalloproteinases are modifiers of huntingtin proteolysis and toxicity in Huntington's disease. Neuron 67, 199–212. 10.1016/j.neuron.2010.06.021 20670829 PMC3098887

[B54] Mizoguchi H. Takuma K. Fukuzaki E. Ibi D. Someya E. Akazawa K. H.. (2009). Matrix metalloprotease-9 inhibition improves amyloid beta-mediated cognitive impairment and neurotoxicity in mice. J. Pharmacol. Exp. Ther. 331, 14–22. 10.1124/jpet.109.154724 19587312

[B55] Moore C. S. Crocker S. J. (2012). An alternate perspective on the roles of TIMPs and MMPs in pathology. Am. J. Pathol. 180, 12–16. 10.1016/j.ajpath.2011.09.008 22033229

[B56] Moore C. S. Milner R. Nishiyama A. Frausto R. F. Serwanski D. R. Pagarigan R. R.. (2011). Astrocytic tissue inhibitor of metalloproteinase-1 (TIMP-1) promotes oligodendrocyte differentiation and enhances CNS myelination. J. Neurosci. 31, 6247–6254. 10.1523/JNEUROSCI.5474-10.2011 21508247 PMC3090636

[B57] Mroczko B. Groblewska M. Barcikowska M. (2013). The role of matrix metalloproteinases and tissue inhibitors of metalloproteinases in the pathophysiology of neurodegeneration: a literature study. J. Alzheimers. Dis. 37, 273–283. 10.3233/JAD-13064 23792694

[B58] Nagase H. (1998). Cell surface activation of progelatinase A (proMMP-2) and cell migration. Cell Res. 8, 179–186. 10.1038/cr.1998.18 9791731

[B59] Nagase H. Visse R. Murphy G. (2006). Structure and function of matrix metalloproteinases and TIMPs. Cardiovasc. Res. 69, 562–573. 10.1016/j.cardiores.2005.12.002 16405877

[B60] Poulsen K. T. Armanini M. P. Klein R. D. Hynes M. A. Phillips H. S. Rosenthal A. (1994). TGF beta 2 and TGF beta 3 are potent survival factors for midbrain dopaminergic neurons. Neuron 13, 1245–1252. 10.1016/0896-6273(94)90062-0 7946360

[B61] Ra H. J. Parks W. C. (2007). Control of matrix metalloproteinase catalytic activity. Matrix. Biol. 26, 587–596. 10.1016/j.matbio.2007.07.001 17669641 PMC2246078

[B62] Ring K. L. An M. C. Zhang N. O'Brien R. N. Ramos E. M. Gao F.. (2015). Genomic analysis reveals disruption of striatal neuronal development and therapeutic targets in human huntington's disease neural stem cells. Stem Cell Reports 5, 1023–1038. 10.1016/j.stemcr.2015.11.005 26651603 PMC4682390

[B63] Sanchez-Capelo A. Colin P. Guibert B. Biguet N. F. Mallet J. (2003). Transforming growth factor beta1 overexpression in the nigrostriatal system increases the dopaminergic deficit of MPTP mice. Mol. Cell. Neurosci. 23, 614–625. 10.1016/S1044-7431(03)00081-2 12932441

[B64] Si-Tayeb K. Monvoisin A. Mazzocco C. Lepreux S. Decossas M. Cubel G.. (2006). Matrix metalloproteinase 3 is present in the cell nucleus and is involved in apoptosis. Am. J. Pathol. 169, 1390–1401. 10.2353/ajpath.2006.060005 17003494 PMC1780186

[B65] Stetler-Stevenson W. G. (2008). Tissue inhibitors of metalloproteinases in cell signaling: metalloproteinase-independent biological activities. Sci. Signal. 1:re6. 10.1126/scisignal.127re6 18612141 PMC2493614

[B66] Tan H. K. Heywood D. Ralph G. S. Bienemann A. Baker A. H. Uney J. B. (2003). Tissue inhibitor of metalloproteinase 1 inhibits excitotoxic cell death in neurons. Mol. Cell. Neurosci. 22, 98–106. 10.1016/S1044-7431(02)00024-6 12595242

[B67] Tejima E. Guo S. Murata Y. Arai K. Lok J. van Leyen K.. (2009). Neuroprotective effects of overexpressing tissue inhibitor of metalloproteinase TIMP-1. J. Neurotrauma. 26, 1935–1941. 10.1089/neu.2009.0959 19469687 PMC2822804

[B68] Tesseur I. Nguyen A. Chang B. Li L. Woodling N. S. Wyss-Coray T.. (2017). Deficiency in neuronal TGF-beta signaling leads to nigrostriatal degeneration and activation of TGF-beta signaling protects against MPTP neurotoxicity in mice. J. Neurosci. 37, 4584–4592. 10.1523/JNEUROSCI.2952-16.2017 28363982 PMC5413189

[B69] Vandenbroucke R. E. Libert C. (2014). Is there new hope for therapeutic matrix metalloproteinase inhibition? Nat. Rev. Drug Discov. 13, 904–927. 10.1038/nrd4390 25376097

[B70] Walker E. J. Rosenberg G. A. (2009). TIMP-3 and MMP-3 contribute to delayed inflammation and hippocampal neuronal death following global ischemia. Exp. Neurol. 216, 122–131. 10.1016/j.expneurol.2008.11.022 19111539 PMC2709713

[B71] Wang C. E. Tydlacka S. Orr A. L. Yang S. H. Graham R. K. Hayden M. R.. (2008). Accumulation of N-terminal mutant huntingtin in mouse and monkey models implicated as a pathogenic mechanism in Huntington's disease. Hum. Mol. Genet. 17, 2738–2751. 10.1093/hmg/ddn175 18558632 PMC2733806

[B72] Weekman E. M. Wilcock D. M. (2016). Matrix Metalloproteinase in blood-brain barrier breakdown in dementia. J. Alzheimers Dis. 49, 893–903. 10.3233/JAD-150759 26599057

[B73] Wellington C. L. Ellerby L. M. Gutekunst C. A. Rogers D. Warby S. Graham R. K.. (2002). Caspase cleavage of mutant huntingtin precedes neurodegeneration in Huntington's disease. J. Neurosci. 22, 7862–7872. 12223539 10.1523/JNEUROSCI.22-18-07862.2002 PMC6758089

[B74] Wellington C. L. Singaraja R. Ellerby L. Savill J. Roy S. Leavitt B.. (2000). Inhibiting caspase cleavage of huntingtin reduces toxicity and aggregate formation in neuronal and nonneuronal cells. J. Biol. Chem. 275, 19831–19838. 10.1074/jbc.M001475200 10770929

[B75] Zhao W. Q. Li H. Yamashita K. Guo X. K. Hoshino T. Yoshida S.. (1998). Cell cycle-associated accumulation of tissue inhibitor of metalloproteinases-1 (TIMP-1) in the nuclei of human gingival fibroblasts. J. Cell. Sci. 111 (Pt 9), 1147–1153. 9547291 10.1242/jcs.111.9.1147

