# Supplementary material for: Altered Expression of Matrix Metalloproteinases and Their Endogenous Inhibitors in a Human Isogenic Stem Cell Model of Huntington's Disease
Source: Front Neurosci. 2018 Feb 5;11:736. doi: 10.3389/fnins.2017.00736 (PMC5807396; doi:10.3389/fnins.2017.00736)
Supplement: Supplementary file 1 [file DataSheet1.docx]

Supplementary Material

Altered Expression of Matrix Metalloproteinases and their Endogenous Inhibitors in a Human Isogenic Stem Cell Model of Huntington’s Disease

Swati Naphade, Alexander Embusch, Kuruwitage Lakshika Madushani, Karen Ring, Lisa M. Ellerby*

*** Correspondence:** Lisa Ellerby: [lellerby@buckinstitute.org](mailto:lellerby@buckinstitute.org)

## Supplementary Figures


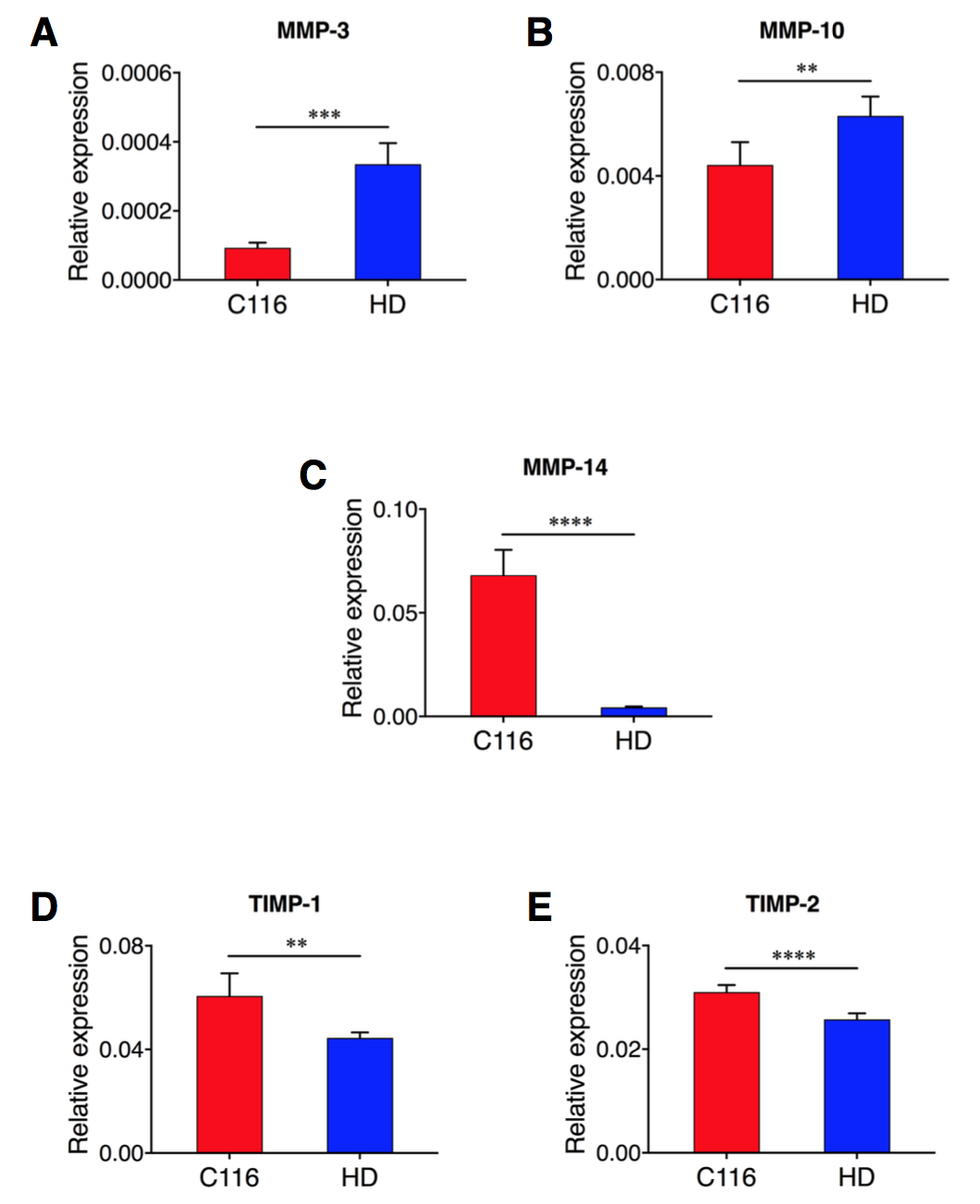


**Supplementary Figure 1.** RT-PCR analysis of MMP/TIMP expression in C116- and HD-NSCs. MMP-3 **(A)** and MMP-10 **(B)** mRNA levels are significantly elevated in HD-NSCs while MMP-14 **(C)** levels are significantly decreased. Both TIMP-1 **(D)** and TIMP-2 **(E)** are significantly downregulated in HD-NSCs. (***p* < 0.01; *** *p* < 0.001; **** *p* < 0.0001; t-test). Error bars represent SD.


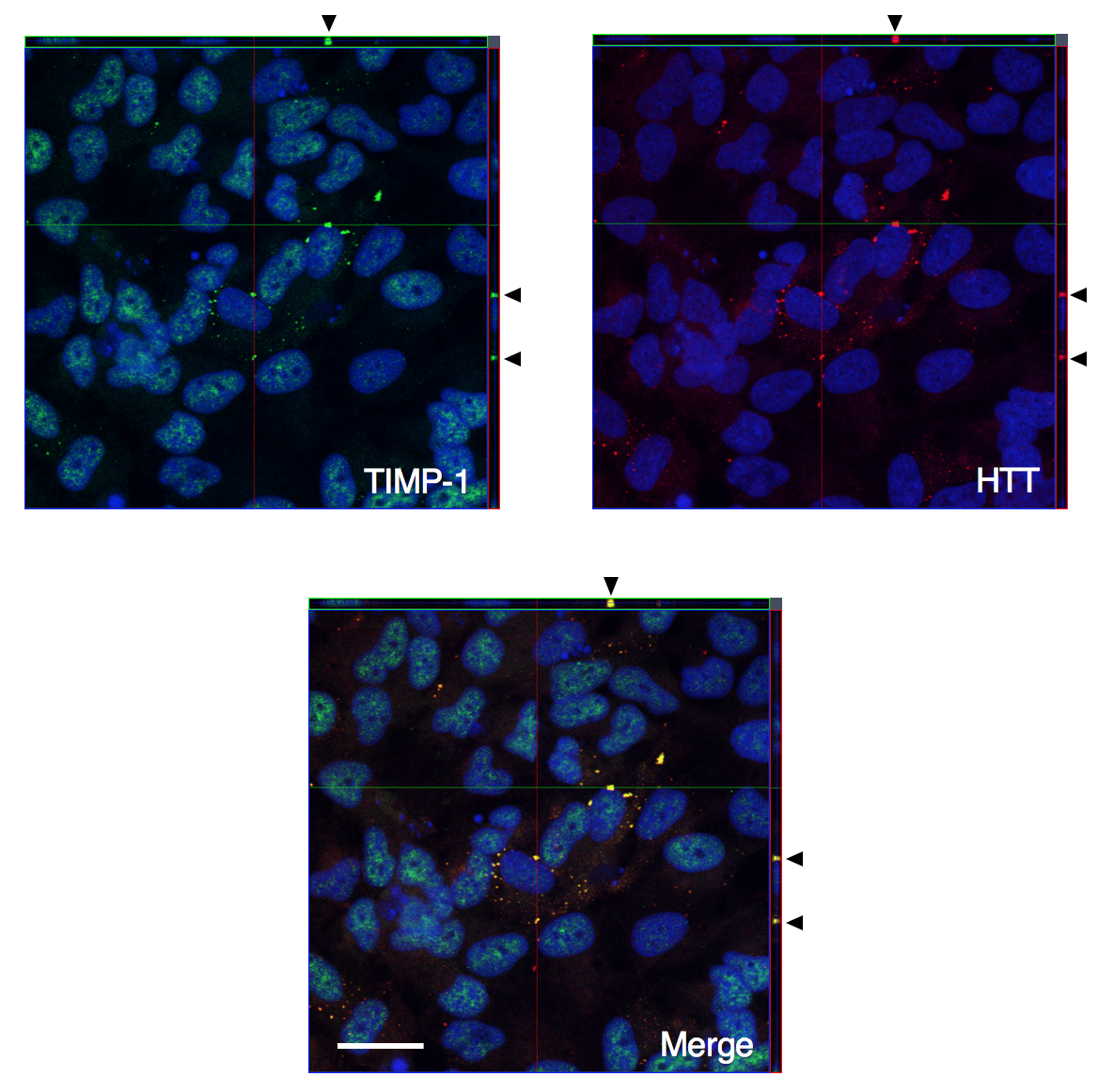


**Supplementary Figure 2.** TIMP-1 coexists with mHTT aggregates in HD-NSCs. Orthogonal XZ and YZ views of a z-stack acquired on a confocal microscope at 63X oil show that TIMP-1 co-exists with mHTT aggregates (black arrowheads). Scale Bar: 25 μm.


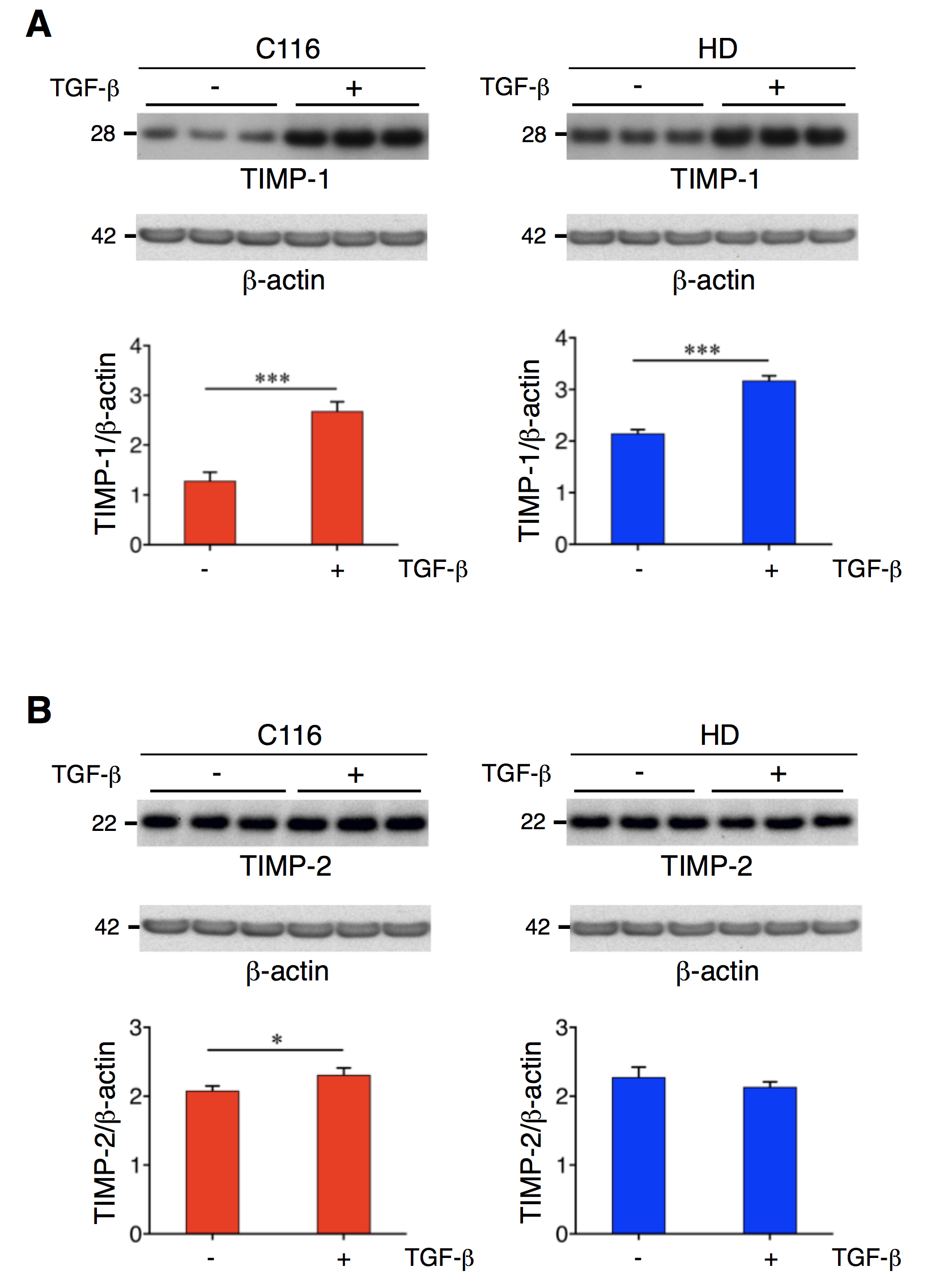


## Supplementary Figure 3. TGF-β treatment regulates TIMP-1 expression. C116- and HD-NSCs were treated with 20 ng/mL TGF-β for 24 h. Western blot analysis reveals that treatment with TGF-β at this higher dose dramatically upregulates TIMP-1 expression (A). An induction in TIMP-1 expression is also observed in C116-NSCs, which is not observed at the lower dose of 10 ng/mL (Figure 5B). Consistent with previous findings (Figure 5C), no changes are observed in the expression levels of TIMP-2 (B). (**p* < 0.05; ****p* < 0.001; t-test). Error bars represent SD.

1. **Tables**

**Table 1.** List of primary and secondary antibodies

**Table 2.** RT PCR analysis - Primers and probes.
